# Biochar production under different atmospheres: an overview

**DOI:** 10.1007/s42773-026-00626-8

**Published:** 2026-07-29

**Authors:** Ondřej Mašek, Wolfram Buss, Liang Wang, Jiacheng Sun, Xutong Wang, Yue Wang, Øyvind Skreiberg

**Affiliations:** 1 UK Biochar Research Centre, School of Geosciences, University of Edinburgh, Crew Building, Alexander Crum Brown Road, Edinburgh, EH9 3FF UK; 2 Research School of Biology, Australian National University, 134 Linnaeus Way, Canberra, ACT 2601 Australia; 3 SINTEF Energy Research, Sem Sælands Vei 11, NO-7034 Trondheim, Norway; 4 School of Environmental Science and Engineering, Tianjin University, Tianjin, 300072 China; 5 Key Laboratory of Green Chemistry and Technology, Ministry of Education, College of Chemistry, Sichuan University, Chengdu, Sichuan 610064 People’s Republic of China; 6 Fenner School of Environment and Society, Australian National University, Canberra, Australia

**Keywords:** Biomass pyrolysis, Biochar, Atmosphere, Carbon dioxide, Steam, Ammonia

## Abstract

The composition of biomass feedstock and pyrolysis parameters, such as temperature, heating rate, and residence time, are key factors that affect the distribution and properties of pyrolysis products and are among the most extensively studied. However, the pyrolysis atmosphere also plays a crucial role in determining process efficiency and product quality. This review examines the effects of various pyrolysis atmospheres on biochar yields and properties, and also considers impacts on pyrolysis gases and liquids. While nitrogen is commonly used in research, the demand for biochar with specific properties like increased porosity and functionality has led to exploring alternative atmospheres such as steam, oxidative, pyrolysis gas, carbon dioxide, methane and ammonia. These alternatives can produce biochar with the desired characteristics in a single step, bypassing the need for multiple modifications. This review provides an overview of these pyrolysis atmosphere options, their applications, advantages, and potential challenges. Future research directions are also identified and highlighted, offering a roadmap for advancing biochar production technology.

**Graphical Abstract:**

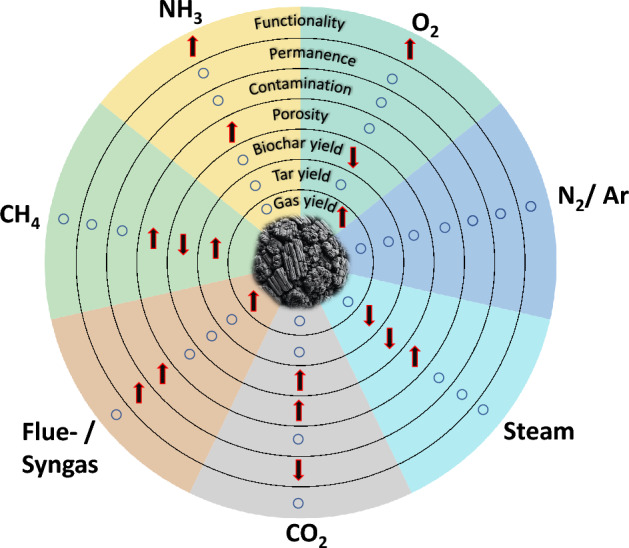

**Supplementary Information:**

The online version contains supplementary material available at 10.1007/s42773-026-00626-8.

## Highlights

- Reactive gases (CO_2_, H_2_O, O_2_, NH_3_) enable one‐step control of biochar yields, porosity and surface chemistry.
- CO_2_ and steam raise surface area, microporosity, and pyrolysis gas/bio‐oil yields (CO_2_ limits tars; steam adds oxygen functionalities).
- NH_3_ (< 400 °C) nitrogenates char to improve cation exchange capacity and catalytic performance.

**Supplementary Information:**

The online version contains supplementary material available at 10.1007/s42773-026-00626-8.

## Introduction

Pyrolysis is the thermo-chemical conversion of biomass in the complete or partial absence of an oxidising medium, resulting in the production of solid char, liquid, and gaseous products. The yield and properties of pyrolytic products and the pyrolysis process efficiency are affected by several process parameters, including pyrolysis temperature, heating rate, residence time, pressure, and atmosphere, in addition to biomass composition. Biochar, a carbon-rich solid co-product of pyrolysis, has gained a lot of interest in the past decade due to its potential role in atmospheric CO_2_ removal, as well as its simultaneous ability to deliver a range of environmental co-benefits (Joseph et al. 2021). For biochar to be effective in storing atmospheric carbon and in delivering potential co-benefits, it is critical to ensure that it possesses the necessary physicochemical properties and is produced sustainably.

Most biochar research published to date used inert atmospheres, most often nitrogen (N_2_), due to its widespread availability, relatively low cost, and good performance. The use of other gaseous atmospheres in the pyrolysis literature is comparably less common but is on an upward trend driven by interest in the production of engineered biochar with specific functionalities. The pyrolysis atmosphere plays a significant role in biomass conversion, affecting heat and mass transfer in the reactor and, in some cases, chemically reacting with pyrolysis products. Besides inert gases (e.g., N_2_ and Ar), reductive (mainly H_2_ and CH_4_), steam, and oxidising gases (O_2_ in sub-stoichiometric ratios) have been employed in published studies, typically to achieve specific effects on the products of pyrolysis.

Another reason for the use of other gases in pyrolysis is to alter and improve the efficiency of the process. Biomass pyrolysis can be an energy-intensive process, as most thermolytic reactions are endothermic until a level of activation energy is exceeded, when the process turns exothermic. It is, therefore, possible to increase the efficiency of the pyrolysis process to increase its economic viability. Pyrolysis of biomass in a reactive atmosphere, such as CO_2_, is gaining interest as CO_2_ not only assists in improving the properties of the produced biochar but also reduces the formation of tar and enhances generation of pyrolysis gas, facilitating energy recovery from this co-product (Kwon et al. 2013a, b; Lee et al. 2017b). In addition, utilization of flue gases from the combustion of the pyrolysis products or from other sources in industrial processes could bring significant advantages, obviating the need for the supply of specific technical gases.

The pyrolysis atmosphere can significantly affect the pyrolysis process and biochar properties. However, so far, no study has assessed these effects across the diverse range of pyrolysis atmospheres employed in both research and practical applications. This review provides an overview of the effects of various pyrolysis atmospheres on biochar production and properties. It discusses the applications, advantages, and challenges of each atmosphere, highlighting the potential for future research and innovation in this field. Such an overview and identified knowledge gaps can catalyse further research and development into pyrolysis atmospheres as a strategic lever to optimize biochar, energy, and chemical production outcomes. The practical value of a given atmosphere depends not on whether it is universally superior to inert pyrolysis, but on which performance objective is being prioritized: retained solid carbon, engineered functionality, gas/liquid upgrading, or process integration.

The coverage allocated to each atmosphere in this review broadly reflects the volume and maturity of the available literature. Sections on CO_2_ and steam are correspondingly more detailed because substantially more primary studies are available, whereas discussions of CH₄ and flue-/pyrolysis gas atmospheres are necessarily more concise owing to the limited number of published investigations. Where appropriate, knowledge gaps for these less-studied atmospheres are explicitly identified to guide future research. To ensure transparency and reproducibility, the next section outlines the semi-systematic search strategy and scope used for study selection and data extraction.

## Search strategy and scope

We conducted a semi-systematic literature search using Google Scholar as the primary engine, complemented by backward and forward citation chaining from seed papers. An adapted funnel strategy was applied: (i) broad scoping with queries combining biochar/char, pyrolysis, and the target process atmosphere (e.g., CO_2_, O_2_/air, CH_4_, NH_3_; including common synonyms such as “oxidative pyrolysis” and “atmosphere-assisted pyrolysis”); (ii) topical filtering to studies that explicitly varied the process atmosphere and reported primary product distributions and/or biochar properties; (iii) inclusion/exclusion: included papers reported operating conditions (temperature, heating rate/residence time, gas composition/flow) and at least one of yields, surface area/porosity, surface chemistry, or functional performance; we excluded post-activation or gasification studies unless a pyrolysis-only comparator under matched conditions was provided, and we used modeling papers and narrative reviews for mechanistic context rather than quantitative tables; (iv) structured extraction of feedstock, reactor/scale, temperature profile, atmosphere composition/flow, yields, and biochar metrics. This approach is transparent but not exhaustive; where reporting heterogeneity limited cross-comparison, we prioritized internally comparable studies and indicated gaps as priorities for future work.

Throughout this review, the term “biochar” denotes the solid carbon-rich product of biomass pyrolysis when produced with the intent of application (e.g., soil amendment, carbon sequestration, or contaminant sorption), following the definition of the International Biochar Initiative. The term “char” is used in mechanistic discussions to refer generically to the solid carbonaceous residue of thermal decomposition, without implying a specific end use, for example, in contexts such as “char,” “secondary char formation,” and “nascent char”. For gaseous products, “pyrolysis gases” refers to the complete gaseous fraction evolved during pyrolysis, encompassing CO_2_, CO, H_2_, CH_4_, and light hydrocarbons.

## Mechanistic primer: how process gases influence pyrolysis chemistry

Pyrolysis proceeds via coupled devolatilisation, condensation/aromatisation, and secondary gas–solid reactions. The process atmosphere modulates radical lifetimes, heat release/uptake, and surface reactions, thereby shifting product distributions and char chemistry.

(1) Devolatilization. Cleavage of glycosidic, ether, and C–C linkages generates volatiles and radical fragments. Oxidizing species (low O_2_/air) can shorten radical lifetimes via abstraction/partial combustion (exothermic), lowering apparent activation barriers and promoting lighter gases at the expense of condensables. CO_2_/H_2_O weakly interact during primary devolatilization but influence secondary cracking via endothermic gasification/reforming of tars and nascent char. H_2_ can cap radicals and suppress cross-linking; effects are modest without catalysts but can temper secondary polycondensation. Inert carriers (e.g., N_2_) mainly provide heat and residence-time control.

(2) Condensation and aromatisation. As temperature rises, polyaromatic domains grow through dehydrogenation, ring fusion, and cross-linking. Mild oxidation (O_2_/air) and etching agents (CO_2_, H_2_O) remove disordered carbon, biasing toward more condensed domains while decreasing solid yield. Hydrogen donors (H_2_, CH₄ cracking at high temperature) can partially stabilize fragments, moderating excessive condensation.

(3) Gas–solid interactions and secondary reforming. Key reactions include Boudouard (C + CO_2_ ⇌ 2CO), steam gasification (C + H_2_O ⇌ CO + H_2_), water–gas shift (CO + H_2_O ⇌ CO_2_ + H_2_), low-level oxidation (C + ½O_2_ → CO/CO_2_), and hydrogenation of surface radicals. These reactions alter porosity (etching creates micro/mesopores), heteroatom content (O/C and H/C decline with increasing severity), and the composition of gases and oils (e.g., reforming decreases oxygenates, increases CO/H_2_). NH_3_ can introduce N functionalities (pyridinic/pyrrolic/quaternary-N) at elevated temperature, affecting surface basicity and adsorption behavior.

Implications. In practice, oxidizing/etching atmospheres tend to lower char yield and raise surface area, whereas hydrogenating/reducing atmospheres temper secondary condensation and can shift volatiles toward lighter, less oxygenated products. The net effect depends on temperature, residence time, feedstock composition, and gas partial pressures; therefore, observed trends should be interpreted alongside these parameters. For a compact mapping of gas-specific reactions, indicative temperature windows, and first-order trends, see SI Table S1.

## Biochar production under inert atmospheres (N_2_, Ar)

Biomass pyrolysis under an inert atmosphere is the most widely studied type of pyrolysis to date, with the vast majority of published research on biomass pyrolysis reporting experiments carried out under an N_2_ atmosphere, followed by Ar. Helium has also been used, but due to its cost and availability, helium is restricted to specialized applications and will, therefore, not be covered in this review. Key reactions and expected product/char trends are summarized in SI Table S1. The use of an inert atmosphere minimises or completely prevents the oxidation of C, as well as several other side reactions (Dong et al. 2022; Fu et al. 2022; Singh et al. 2022; Suriapparao et al. 2022; Xing et al. 2022), resulting in a relatively high yield of biochar and bio-oil. Argon, as a noble gas, is more inert than N_2_, although both gases are sufficiently inert at temperatures commonly used in biochar production, i.e., up to approximately 700 °C.

Despite their similarities, there are also considerable differences in the thermophysical properties of the two gases. This impacts heat and mass transfer in the pyrolysis process and therefore could affect the yield and properties of the resulting products.

Previous work, however, did not observe differences in pyrolysis product yields comparing Ar with N_2_ atmosphere despite differences in molar heat capacity and thermal conductivity of the two gases (Table 1). This was, for example, shown in Bieniek et al. using spent brewer’s grain (Bieniek et al. 2022) or Heuer et al. using coal (Heuer et al. 2016). Research on coal pyrolysis showed that there is a higher characteristic pyrolysis temperature in an N_2_ atmosphere compared to Ar (Lu et al. 2001), while the activation energy and pre-exponential factor are higher in an Ar atmosphere (Lu et al. 2001).

**Table 1:** Thermophysical properties of the common inert gases used for pyrolysis

	N_2_	Ar
Molecular weight	28.013	39.948
Specific gravity (air = 1)	0.967	1.38
Specific volume (m^3^ kg^−1^)	0.872	0.622
Density of liquid at atmospheric pressure (kg m^−^^3^)	808.4	1400
Absolute viscosity (centipoise)	0.018	0.02
Sound velocity in gas (m s^−1^)	353	322
Specific heat (c_p_, cal g^−1^ ^o^C^−1^)	1040	523
Specific heat ratio (c_p_/c_v_)	1.40	1.67
Gas constant (R, J kg^−1^ ^o^C^−1^)	297	208
Thermal conductivity (W m^−1^ ^o^C^−1^)	0.026	0.0172
Boiling point (at saturation pressure 14.7 psia, 760 mm Hg, ^o^C)	− 195.8	− 186
Latent heat of evaporation at boiling point (J kg^−1^)	199,000	163,000
Freezing or melting point at 1 atm (^o^C)	− 210	− 189.2
Latent heat of fusion (J kg^−1^)	25,800	29,759
Critical temperature (^o^C)	− 147	4.87
Critical pressure (MN m^−^^2^)	3.40	
Critical volume (m^3^ kg^−1^)	0.00318	0.00186
Flammable	no	no

Under inert atmospheres such as N_2_ and Ar, pyrolysis proceeds through thermal degradation of biomass macromolecules in the absence of reactive species that form a carbonaceous, highly aromatic framework (Sierra-Jimenez et al. 2023; Vuppaladadiyam et al. 2023). This preserves the molecular identity of pyrolysis intermediates and favors high char and liquid yields but also low surface functionality. Functional groups on the surface of biomass, such as carbonyl, carboxyl and hydroxyl, and methoxyl groups, are mostly cleaved from the carbon molecules and replaced by hydrogen (degree depending on pyrolysis temperature), and an aromatic carbon framework of high stability prevails (Kawamoto 2017; Vuppaladadiyam et al. 2023). The decomposition of cellulose, hemicellulose, and lignin follows well-characterized radical-driven fragmentation and rearrangement mechanisms, including dehydration, depolymerisation, decarboxylation, and cross-linking (Di Blasi 2008). Detailed mechanistic understanding of biomass pyrolysis reactions is reviewed elsewhere (Kawamoto 2017; Lu and Gu 2022; Vuppaladadiyam et al. 2023).

Although Ar and N_2_ differ in thermal conductivity and molar heat capacity, the influence of these gas properties on product distribution is limited due to the dominance of biomass decomposition reactions over heat/mass transfer effects in small-scale reactors (Eseltine 2011). Consequently, inert conditions offer a baseline for studying intrinsic thermal conversion pathways without complicating gas–solid reactions.

## Biochar production under a CO_2_ atmosphere

The concept of CO_2_-assisted pyrolysis for biochar production has been studied extensively, using various lignocellulosic biomasses, sludge and waste materials from different industrial processes (Azuara et al. 2017; Cho et al. 2017; Jung et al. 2016, 2020; Kim et al. 2017, 2021, 2019a; Kończak et al. 2020, 2019; Kwon et al. 2013a, b; Lee et al. 2021; Senneca et al. 2020, 2018). Key reactions and expected product/char trends are summarized in SI Table S1. Serial and parallel reactions either heterogeneously or homogenously take place during the pyrolysis of biomass, which can be roughly grouped into intraparticle/primary pyrolysis and extraparticle/secondary pyrolysis (Senneca et al. 2020; Shen et al. 2017; Yi et al. 2022). Many studies have reported that CO_2_ can act as a reaction medium and be involved in both reaction pathways during biomass pyrolysis. This, subsequently, affects yields and properties of pyrolytic products, and energy conversion efficiency of the pyrolysis process, as shown in Table 2.

**Table 2:** Literature studies discussing the effects of CO_2_ and N_2_ atmospheres on yield of pyrolytic products. The upwards and downwards arrows show higher and lower yields in CO_2_ atmosphere compared to N_2_ atmosphere, respectively

Feedstock	Pyrolysis reactor	Pyrolysis temperature	Pyrolysis atmosphere (vol% CO_2_)^a^	Biochar (wt%)	Liquid (wt%)	Gas (wt%)	Reference
Oil palm fibres	Quartz tubular reactor	400 °C	0	37.2	27.2	36.1	Chen and Lin (2016)
		400 °C	100	37.4↑	25.5↓	37.1↑	
	Quartz tubular reactor	450 °C	0	36.3	26.6	37.2	
		450 °C	100	37.2↑	25.8↓	37.6↑	
	Quartz tubular reactor	500 °C	0	31.5	32.1	37.3	
		500 °C	100	32.3↑	31.5↓	37.5	
Coconut husk	Quartz tubular reactor	675 °C	0	32.9	32.97	37.27	Kwon et al. (2024)
		675 °C	100	37.3↑	29.7↓	33.10↓	
Microalgae	Quartz tubular reactor	600 °C	0	31.3	44.4	32.3	Jung et al. (2016)
		600 °C	100	32.3↑	43.5↓	26.7↓	
Red pepper stalk	Quartz tubular reactor	600 °C	0	21.5	31.8	46.8	Lee et al. (2017b)
		600 °C	100	22.9↑	21.0↓	56.1↑	
Euglena gracilis	Fixed-bed reactor	720 °C	0	60.4	10.7	28.9	Jung et al. (2019)
		720 °C	100	64.1↑	7.2↓	28.7↓	
Paper sludge	Fixed-bed reactor	720 °C	0	43.2	56.5	0.5	Lee et al. (2017a)
		720 °C	100	41.8↓	36.2↓	22.0↑	
Crude oil sludge	Fixed-bed reactor	700 °C	0	5.2	20.6	74.2	Kim et al. (2019a, b, c, d)
		700 °C	100	5.1↓	14.6↓	80.3↑	
Goat excreta residue	Fixed-bed reactor	720 °C	0	24.1	40.3	35.6	Kim et al. (2019a)
		720 °C	100	23.9↓	36.1↓	40.6↑	
Rice straw	Fixed-bed reactor	720 °C	0	37.5	55.1	7.5	Jung et al. (2019)
		720 °C	100	38.3↑	54.6↓	7.1↓	
Peat moss	Fixed-bed reactor	650 °C	0	40.6	16.1	43.3	Lee et al. (2020a, b, c)
		650 °C	100	39.7↓	15.1↓	45.2↑	
Tobacco leaf waste	Fixed-bed reactor	720 °C	0	34.7	15.5	49.8	Lee et al. (2021)
		720 °C	100	31.5↓	14.5↓	54.0↑	
Tobacco stick waste	Fixed-bed reactor	720 °C	0	25.7	18.4	55.9	
		720 °C	100	24.2↓	13.7↓	62.2↑	
Seaweed	Quartz tubular reactor	700 °C	0	7.0	48.0	45.0	Kwon et al. (2013a, b)
		700 °C	100	8.0↑	23.0↓	69.0↑	
Corn stover	Quartz tubular reactor	700 °C	0	23.0	39.0	38.0	Choi et al. (2020)
		700 °C	100	27.0↑	14.0↓	59.0↑	
Oak wood	Quartz tubular reactor	700 °C	0	22.0	39.0	39.0	Kwon et al. (2013a, b)
		700 °C	100	24.0↑	17.0↓	59.0↑	
Orange peel	Quartz tubular reactor	700 °C	0	27.0	44.6	28.4	Kim et al. (2024)
		700 °C	100	31.3↑	38.9↓	29.8↑	
Cellulose	Quartz tubular reactor	700 °C	0	32.0	31.0	37.0	Kwon et al. (2013a, b)
		700 °C	100	33.0↑	25.0↓	42.0↑	

^a^0 means pyrolysis experiments were conducted under an inert atmosphere with 100% N_2_ or Ar

The presence of CO_2_ can affect the thermal degradation of biomass and the extent of impacts is considerably related to pyrolysis parameters (e.g., temperature) and feedstock properties (Bora et al. 2024; Lee et al. 2025a). Carbon dioxide-assisted pyrolysis can promote the cracking of volatile organic compounds (VOCs)/tar and affect the chemical compositions of the bio-oil. This contributes to a shift of carbon in the pyrolysis liquid products into carbon monoxide-rich pyrolysis gas (Choi et al. 2025; Kim et al. 2025a, b; Lee et al. 2025a). The carbon monoxide-rich pyrolysis gas can be used for power and heat generation. The CO_2_ emitted from the combustion of pyrolysis gas can be used as the atmosphere for pyrolysis. This enables lowering the carbon footprint from the overall pyrolysis process (Choi et al. 2025; Kim et al. 2025a, b; Lee et al. 2025a, b, c; Lee et al. 2025a, b). For enhancing the conversion of the biomass into products with desired compositions for practical use, studies were also conducted on catalytic pyrolysis using CO_2_ as a purge gas (Kim et al. 2025a, b; Lee et al. 2025a, b, c; Lee et al. 2025a, b). The use of a catalyst enhances the reactions of CO_2_ with volatiles derived from the degradation of biomass, increasing the yield of CO in the produced pyrolysis gas. The intensity and pathways of reactions between CO_2_ with both charring biomass particles and volatile products are considerably related to pyrolysis temperature and feedstock type. These will consequently impact the yield and composition of the generated pyrolysis oil and gas products, as described in Sects. 5.1–5.3.

### Yield and composition of pyrolysis liquids

Table 2 provides the yield of pyrolytic products from studies focused on tar reduction when using CO_2_ as a pyrolysis carrier gas. Different types of biomass with various properties have been studied, such as micro and macro algae (Jung et al. 2016; Lee et al. 2017b), wood (Choi et al. 2020; Kim et al. 2017, 2021), sawdust (He et al. 2020), rice straw (Jung et al. 2020), red pepper stalk (Lee et al. 2017b), swine manure (Kwon and Castaldi 2012; Lee et al. 2017a, b, c, d, e, f), cow manure (Lee et al. 2020a, b, c), peat (Lee et al. 2017e), spent coffee grounds (Cho et al. 2017; Kim et al. 2019b), paper sludge (Cho et al. 2017; Lee et al. 2017c), crude oil sludge (Kim et al. 2019a, b, c, d), tobacco waste (Lee et al. 2021), and grass (Kim et al. 2021, 2019c). The addition of CO_2_ reduces tar formation during biomass pyrolysis, while increasing the gas and char yield due to the cracking of tar into gas and secondary char. Elevated pyrolysis temperatures, i.e., temperatures above approximately 700 °C, however, can result in reduced biochar yield due to CO_2_ gasification and, therefore, need to be avoided (further discussed in biochar section below). The magnitude of tar reduction varies depending on the type of biomass feedstock (Lee et al. 2017d). For example, 70% tar reduction was observed from the CO_2_ pyrolysis of seaweed, whereas only 23% tar reduction was observed for paper mill sludge (Lee et al. 2017c). Two main mechanisms were identified: 1) expediting thermal cracking and conversion of the VOCs derived from the pyrolysis of biomass; and 2) direct homogenous reaction between CO_2_ and VOCs via gas phase reactions.

**Fig. 1:**
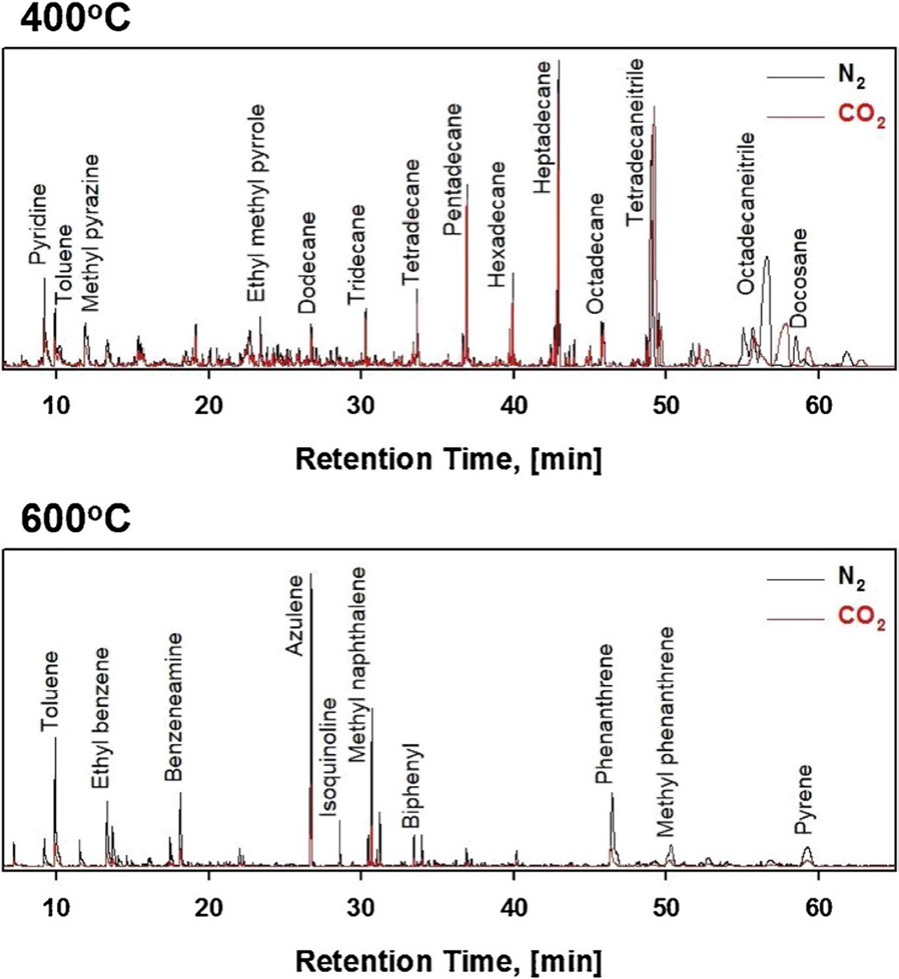
Comparison of pyrogenic products from pyrolysis of swine manure in N_2_ and CO_2_ at 400 and 600 °C (J. Lee et al. 2017d)

The addition of CO_2_ also influences the content of chemical species in the pyrolytic liquid products (Lee et al. 2020a, b, c). Figure 1 shows a representative chromatogram of pyrolytic oil from slow pyrolysis of swine manure in N_2_ and CO_2_ at 400 and 600 °C (Lee et al. 2017b). The major chemical species are labelled, and peak areas of each chemical species indicate the concentration in the liquid collected from CO_2_-aided pyrolysis of swine manure. The variety of chemical species in the pyrolytic oil produced from CO_2_ pyrolysis of this raw material is lower than when produced in N_2_, especially for pyrolytic oil produced at 600 °C. Moreover, both the magnitude and the areas of the peaks related to chemical species detected from pyrolytic oil from CO_2_ pyrolysis are considerably lower than those produced in the presence of N_2_. Similar results regarding changes in yield and composition of pyrolysis oil have been reported in other studies. Jung et al. conducted slow pyrolysis of microalgae (*Microcystis aeruginosa*) in CO_2_ and N_2_ atmosphere by using a fixed-bed reactor (Jung et al. 2016). The pyrolytic oil generated from the decomposition of the microalgae was analyzed by a GC/TOF–MS. The analysis results showed that the content of heavy molecular chemical species in the pyrolysis oil decreased when using CO_2_ as carrier gas (Lee et al. 2020a, b, c; Jung et al. 2016). During pyrolysis of coffee grounds, the yield of pyrolysis oil was lower under CO_2_, reaching only 40–60% of the yield obtained under N_2_ (Kim et al. 2019b), and composition differences of pyrolytic oil were detected. From another study, similar results were observed using goat manure—a reduction in pyrolysis oil from 40.3% in N_2_ to 36.1% in CO_2_ (Kim et al. 2019a) and reduced formation of heavy chemical species in a CO_2_ atmosphere. Such a decrease in the formation of heavy chemical species is mainly related to gas-phase reactions between CO_2_ and VOCs, and CO_2_ expediting thermal cracking of VOCs formed during pyrolysis. The change of yield and composition of pyrolytic oil from CO_2_-aided pyrolysis can be mainly related to potential reactions of CO_2_ with volatile compounds released from the decomposition of the biomass particles. During the CO_2_-aided pyrolysis, CO_2_ can be involved in the defragmentation of the VOC, which generates compounds that repolymerize into different final products than those generated in the presence of N_2_. In addition, with the presence of CO_2_, more intensive thermal cracking of the VOC via dehydrogenation can take place, which is more evident as the pyrolysis is conducted at a higher temperature (Kim et al. 2024). This is generally accompanied by an increase in the yield of gas products. In addition, the tar cracking also inhibits the formation of secondary char. Therefore, the char yields from CO_2_-aided pyrolysis at temperatures above 600 °C can be slightly lower than those obtained from pyrolysis in an N_2_ atmosphere. Furthermore, the interactions of CO_2_ with VOC affect the composition of the pyrolytic oil. Less oxygenated compounds, including benzene derivatives and phenolic compounds, as indicated in Fig. 1, were detected in the pyrolytic oil derived from the CO_2_-aided pyrolysis (Lee et al. 2017d).

### Yield and composition of pyrolysis gases

As discussed above, the added CO_2_ can expedite the thermal cracking of VOCs and directly react with VOCs, which causes a shift of C distribution from pyrolysis oils to pyrolysis gases. At elevated pyrolysis temperatures, CO_2_ can also facilitate gasification reactions of biochar further increasing gas production. Consequently, several studies report increased gas yield during pyrolysis in a CO_2_ atmosphere compared to a N_2_ atmosphere at temperatures above 450 °C (Cho et al. 2015; Choi et al. 2020; Jung et al. 2019; Lee et al. 2017b, 2017c).

**Fig. 2:**
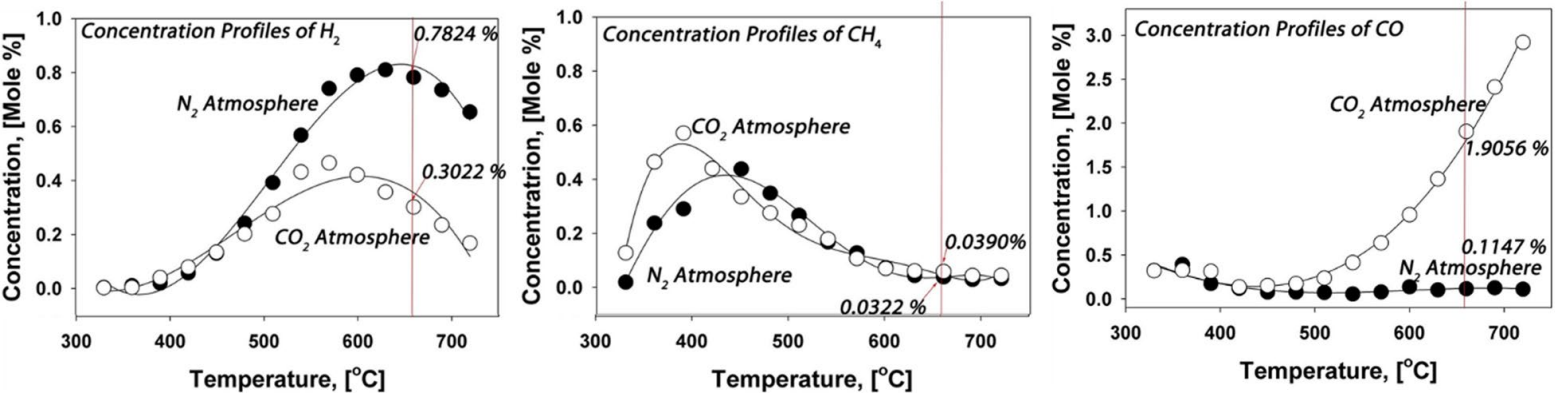
Concentration profiles of the major pyrolytic gases evolved from the thermal degradation of spent coffee grounds in N_2_ and CO_2_ environments (Cho et al. 2015)

Pyrolysis in a CO_2_ atmosphere alters the distribution of individual gaseous products, as shown in Fig. 2, pyrolysing spent coffee grounds in N_2_ and CO_2_ environments (Kim et al. 2019b). It demonstrates enhanced generation of CO as the temperature exceeds 450 °C, with a further sharp increase of CO generation above 550 °C. The concentration of CO at 660 °C in the presence of CO_2_ was ∼17 times higher than in an N_2_ atmosphere, with a further increase at a temperature of 720 °C (∼30 times higher) (Cho et al. 2015). Similar findings were reported investigating the effect of CO_2_ on CO yield during the pyrolysis of various biomass, including oak wood, where the generation of CO was ∼12 times higher at 600 °C in a CO_2_ atmosphere compared to a N_2_ atmosphere (Choi et al. 2020; Lee et al. 2017d, 2018; Lee et al. 2017a, b, c, d, e, f; Cho et al. 2017). A study using paper mill sludge and a temperature range of 450 to 720 °C demonstrated that the CO concentration in the pyrolysis gas was proportional to the amount of CO_2_ used for pyrolysis (Lee et al. 2017c).

The yields and release of H_2_ and CH_4_ also differ during CO_2_-assisted pyrolysis of various biomass, but to a smaller extent than CO in comparison to those generated from pyrolysis in N_2_ atmosphere. The concentration of H_2_ and CH_4_, for example, increased by a factor of 4 and 7, respectively, during the pyrolysis of powdered cellulose and xylan at 680 °C, whereas the concentration of CO was enhanced by a factor of 10 (Kwon et al. 2013a, b). Such promotion of H_2_ and CH_4_ release from pyrolysis of corn stover was also reported in the same study (Kwon et al. 2013a, b). Some studies, however, showed that employing CO_2_ during pyrolysis (of swine manure and corn stover) increased the formation of CO, which was accompanied by suppression of H_2_ (Cho et al. 2015; Lee et al. 2017d). Figure 2 shows the release behavior of H_2_ as reported by Lee et al. (2017d). The suppression effect was more evident as the pyrolysis temperature exceeded a certain value, being 500 °C in this study (Cho et al. 2015). The suppression of H_2_ can partially be related to the dehydrogenation of VOC in the CO_2_ atmosphere during the pyrolysis process. The extents of the suppression effect of CO_2_ on the formation of H_2_ depend on the type of biomass and reaction conditions (Cho et al. 2017; Kim et al. 2021; Lee et al. 2020a, b, c). However, more detailed studies are still needed to identify and elucidate the interactions of CO_2_ with VOCs from the decomposition of biomass and their impacts on the formation of gas species.

The seemingly contradictory behavior of H_2_ yields under CO_2_ atmospheres can be partially reconciled by considering temperature-dependent competition between two pathways. Below ~ 500 °C, CO_2_ promotes dehydrogenation of VOCs, which generates additional H_2_ and can increase H_2_ yields relative to N_2_ (Kwon et al. 2013a, b). Above ~ 500 °C, the reverse water–gas shift reaction (CO_2_ + H_2_ → CO + H_2_O) becomes significant and consumes H_2_, resulting in lower net H_2_ yields (Cho et al. 2015). The temperature at which suppression dominates depends on feedstock composition—specifically, feedstocks that generate H_2_-rich volatiles (e.g., cellulose) may show a higher crossover temperature than those producing CO-rich volatiles (e.g., lignin-rich biomass). Additionally, the residence time and CO_2_ concentration affect the extent to which the reverse water–gas shift equilibrium is approached. These factors collectively explain why some studies report enhancement and others report suppression of H_2_ yields, and underscore the need for systematic parametric studies.

In summary, using CO_2_ as a pyrolysis medium can enhance the generation of pyrolysis gas and CO because of homogeneous and heterogeneous reactions of CO_2_ with gases, tars, and char ( Kwon et al. 2013a, b; Shen et al. 2017).

### Yields and properties of biochar

Studies have been conducted to investigate interactions between biomass feedstock and CO_2_ during biochar formation and the impacts of CO_2_ on reaction chemistry. The influence of CO_2_ on the yield of products from slow pyrolysis of biomass is considerably related to reaction conditions and properties of the feedstock. Depending on the pyrolysis temperature, a CO_2_ atmosphere can increase, decrease or have no effect on biochar yield compared to an inert atmosphere.

At medium temperature range (300–600 °C), the presence of CO_2_ might favor the formation of biochar and liquid products, which hinders the generation of gases. CO_2_ purged into the reactor together with that generated during decomposition of the feedstock results in high CO_2_ partial pressure inside the char matrix and in the vicinity of biomass particles. This constrains the diffusion of small molecules out of the biomass particles. Therefore, the tendency of repolymerization of these products on the surface of charring particles is high, leading to an increase in the yield of biochar under a CO_2_ atmosphere in comparison with N_2_. Slightly lower biochar yields, however, were obtained from slow pyrolysis of certain biomass under CO_2_ in comparison to N_2_ atmosphere, such as wheat straw at 500 °C and oil palm fiber at 450 °C. It indicates that the impacts of CO_2_ on the synthesis of biochar at temperatures below 600 °C depend upon the type of biomass material. More systematic studies are needed to identify and elucidate interactions of CO_2_ with biomass and formation of biochar and by-products upon change of feedstock and operating conditions.

In the higher temperature range (600–800 °C), CO_2_ influence on the yield of biochar from slow pyrolysis of biomass materials is also diverse. When the temperature exceeds ~700 °C, more intensive interactions of CO_2_ with both solid char and volatiles take place and in comparison to pyrolysis in N_2_ atmosphere, lower biochar yields are observed as demonstrated for pepper stalks, swine manure, municipal solid waste biomass fraction, peat, tobacco waste, food waste, oil palm fiber and paper mill sludge (Chen and Lin 2016; Lee et al. 2017a, b, c, d, e, f; Lee et al. 2021; Lee et al. 2020a, b, c). This enhanced biomass decomposition at elevated temperatures in a CO_2_ atmosphere was attributed to the Boudouard reaction of CO_2_ with biochar-C, which increases gas production through gasification and enhances devolatilization, producing CO from the biochar-C. Of the three main biomass constituents, only hemicellulose degradation was substantially accelerated by CO_2_ addition to the pyrolysis atmosphere (Lee et al. 2017b).

In addition to the yield of biochar, CO_2_ can affect the physicochemical properties of the produced biochar, such as the development of the carbonaceous structure of the biochar in several ways. The effect of CO_2_ on the surface area of biochar has been investigated in several studies with data on specific surface area summarized in Table 3 (Ahmad et al. 2021; Cho et al. 2017; Kim et al. 2019a, b, c, d; Kończak et al. 2019; Kwon and Castaldi 2012; Lee et al. 2017d, 2017b). The enhancement of biochar surface area due to the presence of CO_2_ is consistent for most biomass feedstocks, except for biochar from feedstocks with a high ash content, such as sewage sludge, where effects are less pronounced or not visible (Table 3). In comparison to an inert gas atmosphere, the absolute enhancement of the BET surface area of biochar produced in CO_2_ was clear, for pyrolysis with both low and high heating rates. Such enhancements are more evident for biochar produced at a pyrolysis temperature higher than 600 °C, as indicated by the data summarized in Table 3. Such studies also report increased pore volume in biochar produced in a CO_2_ atmosphere (Cho et al. 2015).

**Table 3:** BET specific surface area (SSA) of biochar produced in a CO_2_ and N_2_ atmosphere summarised from the literature. The upwards and downwards arrows show higher and lower SSA in CO_2_ atmosphere compared to N_2_ atmosphere, respectively

Feedstock	Pyrolysis temperature	Pyrolysis atmosphere (vol% CO_2_)	BET SSA (m^2^ g^−1^ N_2_)	Reference
Sewage sludge	500 °C	0	69.70	Kończak et al. (2019)
		100	71.10↑	
Sewage sludge	600 °C	0	75.50	
		100	74.70↓	
Sewage sludge	700 °C	0	89.20	
		100	83.50↓	
Red pepper stalk	600 °C	0	32.46	Lee et al. (2020a, b, c)
		100	109.15↑	
	650 °C	0	85.43	
		100	93.04↑	
Rice straw	400 °C	0	4.99	Jung et al. (2020)
		100	4.65↓	
Rice straw	600 °C	0	24.43	
		100	303.73↑	
Grass	680 °C^a^	0	6.58	Kim et al. (2019c)
		100	21.25↑	
Grass	680 °C^b^	0	11.66	
		100	24.67↑	
Oak wood	680 °C^a^	0	231.15	Kim et al. (2019c)
		100	463.58↑	
Oak wood	680 °C^b^	0	292.99	
		100	386.76↑	
Cellulose	680 °C^a^	0	507.53	Kim et al. (2019c)
		100	506.94↓	
Cellulose	680 °C^b^	0	504.55	
		100	515.04↑	
Xylan	680 °C^a^	0	340.43	Kim et al. (2019c)
		100	402.35↑	
Lignin	680 °C^a^	0	299.97	Kim et al. (2019c)
		100	325.85↑	
Lignin	680 °C^b^	0	360.31	
		100	402.35↑	
Kudzu vines	700 °C	0	4.13	Lee et al. (2025c)
		100	144.22↑	
Brewer’s spent grain	700 °C	0	15.05	Kim et al. (2025a, b)
		100	29.29↑	
Cattle manure	700 °C	0	37.31	Yoon et al. (2024)
		100	98.47↑	
Furfural residues	600 °C	0	274.41	Liu et al. (2024)
		100	329.21↑	
Fruit peel waste	600 °C	0	2.87	Kim et al. (2024)
		100	398.07↑	

^a^ produced through pyrolysis with a heating rate of 10 °C min^−1^

^b^ produced through pyrolysis with a heating rate of 50 °C min^−1^

The total and fixed C content of pepper stalk biochar produced in a CO_2_ atmosphere at 600 °C, however, was higher compared to an inert atmosphere (total C: 86.6 vs. 83.8% and fixed carbon: 66.1 vs 62.1%) (Lee et al. 2017d). The opposite, i.e., a lower biochar C content, was observed during pyrolysis of sewage sludge at 500–700 °C in a CO_2_ atmosphere (Kończak et al. 2020). Interestingly, the effect of a CO_2_ atmosphere on the C content of oil palm fiber biochar, relative to an N_2_ atmosphere, depended on the pyrolysis temperature and whether the biomass was pelleted (Chen and Lin 2016). The C content was lower in oil palm fiber biochar produced in a CO_2_ atmosphere (compared to N_2_) when the biomass was not pelleted, and tended to be higher (compared to N_2_) when the biomass was pelleted, highlighting that the reactions with CO_2_ differ even within the same biomass type (Chen and Lin 2016). In a study testing CO_2_-assisted pyrolysis in the temperature range 650–850 °C, no effect on biochar’s thermal stability was recorded, but decreased chemical stability (Xu et al. 2021). Given these variable responses and in the absence of a detailed mechanistic understanding that explains such effects, the response of C content and stability to the addition of CO_2_ to the pyrolysis atmosphere cannot be generalized.

Several factors may explain these divergent responses. First, feedstocks with high ash content (e.g., sewage sludge) contain catalytic minerals (K, Ca) that accelerate the Boudouard reaction (C + CO_2_ → 2CO), preferentially consuming amorphous carbon and lowering total C content. In contrast, lignocellulosic feedstocks with lower ash (e.g., pepper stalks) may experience enhanced cross-linking and repolymerization under CO_2_ at moderate temperatures, increasing fixed C. Second, the pelleting effect observed for oil palm fiber (Chen and Lin 2016) suggests that mass-transfer limitations play a critical role: pellets restrict volatile egress, increasing secondary char formation under CO_2_ and raising C content, whereas unpelleted biomass allows easier volatile release and greater exposure of the carbon matrix to CO_2_ etching. Third, temperature thresholds are important. Below ~600 °C, CO_2_ may primarily act as an inert diluent or mild repolymerization promoter, whereas above ~600 °C, the Boudouard reaction becomes thermodynamically and kinetically significant, leading to net carbon loss. Future studies should systematically vary ash content, particle size/density, and temperature within single feedstock types to disentangle these effects.

During pyrolysis of biomass, CO_2_ participates in heterogeneous reactions and, with carbonaceous char, drives structural and morphological transformations in the resulting biochar. These reactions disrupt hydrogen-bearing structures and cleave low-molecular-weight functional groups (Choi et al. 2018), weakening the interaction between hydrogen and the char matrix and thereby promoting the generation of H radicals on the biochar surface (Kończak et al. 2019). The liberated H radicals subsequently react with other free radicals produced from the fragmentation of macromolecular char structures, leading to volatile formation (Cho et al. 2015; Jung et al. 2019). Overall, CO_2_ facilitates the cracking of tarry compounds both in the gas phase and within the char matrix, suppressing polymerization processes and yielding biochar with higher microporosity and surface area (Chen and Lin 2016).

### Summary and conclusion—CO_2_

Utilization of CO_2_ as a pyrolysis medium has multiple effects on thermal decomposition behavior of the biomass, reaction chemistry of decomposition pathways, and yield and properties of final products. The product yields included in Table 2 show a higher yield of pyrolysis gases with a lower yield of pyrolysis liquids for the experiments conducted in a CO_2_ atmosphere. CO_2_, as a pyrolysis medium and also a reaction agent under certain pyrolysis conditions, can interact with the volatile organic compounds derived from the decomposition of the biomass. This leads to the transformation of VOC into smaller molecular species and gases that are not condensable and remain as gas products. Interactions between the CO_2_ and VOC also cause changes in the composition of pyrolytic oil. Certain chemical compounds are depleted in the pyrolytic oil from CO_2_-aided pyrolysis, in comparison to those obtained from pyrolysis of the same feedstock in N_2_ atmosphere. Such changes can affect further upgrading and utilization of the pyrolytic oil from the CO_2_-aided biomass pyrolysis process. The impacts of CO_2_ on the properties of the produced biochar are more evident as the temperature exceeds, for example, 600 °C. With further increase of the CO_2_-aided pyrolysis temperature above this value, biochar specific surface area and porosity can be larger, due to more intensive reactions between CO_2_ and VOC and possible reactions of CO_2_ with carbon. Therefore, the impacts of CO_2_ on the properties of biochar need to be carefully considered when selecting and determining further application of the biochar.

The enhanced surface area and porosity of biochar produced in a CO_2_ atmosphere make it particularly suitable for applications requiring high adsorption capacity, such as soil amendment for nutrient retention, remediation of contaminated soils and waters, and as a sorbent for heavy metals and organic pollutants (Cho et al. 2015). The increased aromaticity and thermal stability further support its use in long-term carbon sequestration strategies. The tunability of these properties through process parameters enables the targeted production of biochar for environmental and agricultural applications, positioning CO_2_-assisted pyrolysis as a promising approach for sustainable waste management and the production of value-added biochar.

CO_2_ pyrolysis of biomass has received much interest, and extensive experimental research has been conducted; however, the mechanisms responsible for the observed effects are not fully understood. Systematic experimental studies with in situ monitoring and characterization of intermediate and final products are critical for investigating these mechanisms. The studies reviewed above are mostly carried out based on conventionally heated processes using fixed-bed reactors. It is, therefore, interesting to study CO_2_ pyrolysis behaviors and product formation for biomass at different process parameters (i.e., heating rate, residence time and pressure) in different types of reactors. Such studies are important for the dedicated production of engineered biochar through CO_2_ pyrolysis for high-value products. Process simulation is also important in developing the CO_2_ pyrolysis process, considering factors such as feedstock type, pyrolysis conditions and CO_2_ concentrations. The simulations can be used for assessing and optimizing operation conditions and process performance as well.

It is possible to integrate the CO_2_-pyrolysis process with other industrial processes that produce CO_2_ and heat, e.g., oxyfuel combustion. This would reduce heat waste, allow the utilization of CO_2_, and decrease the carbon footprint for both the biochar production process and the industry that supplies the CO_2_.

## Biochar production under steam atmosphere

The presence of steam during biomass pyrolysis is almost inevitable, either due to its release from moisture contained in biomass or its generation by pyrolysis reactions. Depending on the moisture content and composition of biomass, the amount of steam in the reactor can vary considerably, and it can be difficult to distinguish the role of steam in the reactions. This section, however, focuses on dedicated steam pyrolysis, where steam is introduced as the main carrier/purge gas rather than other inert or reactive gases. Key reactions and expected product/char trends are summarized in SI Table S1.

Pyrolysis under steam atmosphere has been scarcely studied in the literature, with most published research focusing on pyrolysis in fixed-bed reactors (Önal et al. 2017, 2011; Pütün et al. 2006), and a smaller number of studies using a fluidized bed reactor (Kantarelis et al. 2013) and catalytic steam pyrolysis (Ateş et al. 2005; Kantarelis et al. 2014; Pütün et al. 2008).

Steam acts as a mild oxidizing and gasifying agent during pyrolysis, reacting with biochar carbon via steam gasification reactions (C + H_2_O → CO + H_2_), which selectively remove carbon from the char surface and open blocked pores. This process removes tarry deposits and volatile residues, enhancing microporosity and total pore volume. The interaction of steam with biochar also introduces oxygen-containing functional groups, such as hydroxyl and carbonyl species, via partial oxidation, which can increase surface polarity and adsorption sites. Kinetic studies demonstrate that steam partial pressure and temperature strongly influence the gasification rate, with higher steam pressures accelerating char reactivity and pore development.

The pyrolysis temperature determines the level of interaction of steam and pyrolytic reactions. Low to moderate temperatures (< 500 °C) did not affect yields and composition of volatiles compared to a N_2_ atmosphere but at 700 °C steam takes part in the pyrolysis reactions and changes the reaction mechanism, product yields and their properties, similar to gasification reactions demonstrated with woody biomass and cellulose (6 g biomass and 0.25 g s^−1^ steam flow) and apple waste (100 g biomass with 8 dm^3^ min^−1^ steam flow) (Barszcz et al. 2024; Gargiulo et al. 2018; Wang et al. 2020).

### Yield and composition of pyrolysis liquids and gases

Using steam in the pyrolysis atmosphere increases the formation of liquid products, decreasing char and gas yields when compared with pyrolysis using inert gases or CO_2_ (Özbay et al. 2008) (Mengxia et al. 2018; Ouyang et al. 2020; Qing et al. 2019; Tian et al. 2021; Yang et al. 2012). This is, in part, a result of steam gasification reactions, which promote the conversion of char into H_2_-rich gas.

The steam atmosphere has a significant impact on the yield and properties of bio-oil as an important co-product of biomass pyrolysis. Several studies showed that, compared to pyrolysis under an inert atmosphere, pyrolysis in a steam atmosphere increases the yield of bio-oils, especially the aliphatic fractions, and reduces the contents of organic acids (Cheng et al. 2017; Mellin et al. 2015; Minkova et al. 2001). The steam not only removes the primary products from the pyrolysis reactions but also reacts with the products and promotes the evaporation of oils from the biomass and reduces the formation of solid char (Özbay et al. 2008). This results in a change in bio-oil composition as secondary cracking reactions are reduced and the composition of the bio-oil is improved by decreasing the content of acids and increasing the content of aliphatic compounds, as well as the H/C ratio and the heating value (Ateş et al. 2005; Garcia et al. 2008; Kantarelis et al. 2014; Önal et al. 2011; Pütün et al. 2008).

### Yield and properties of biochar

Steam removes coke from the char surface, which decreases secondary char formation reactions (Wang et al. 2021) and hence biochar yields as already discussed above. This reaction could also affect biochar C stability. The fixed C content decreased in a steam environment when pine was pyrolyzed at 600 °C; however, no change was observed at 500 °C (Fernandez et al. 2022). The H/C and O/C ratios, also indicators of C stability, were not affected by the steam atmosphere, indicating minimal effect of steam atmosphere on biochar stability (Fernandez et al. 2022). However, steam pyrolysis can also enhance aromatic ring condensation in biochar, add oxygen-containing functional groups (Mengxia et al. 2018; Qing et al. 2019) and create a disordered char structure (Ouyang et al. 2020). The effects of these reactions could either increase (higher degree of aromatization) or decrease (more oxygen-functional groups and disordered char structure) biochar C stability. This contradiction was demonstrated in a study using steam pyrolysis over a temperature range of 650–850°C, which showed no effect on thermal stability but a weakening of biochar's chemical stability (Xu et al. 2021). Overall, due to the limited number of studies specifically investigating the carbon stability of biochar produced in a steam atmosphere, it is not possible to draw clear conclusions.

The apparently contradictory evidence on steam effects on biochar stability likely reflects competing mechanisms operating at different temperatures. At moderate temperatures (≤ 500 °C), steam interaction with the char surface is limited, and H/C and O/C ratios remain largely unaffected (Fernandez et al. 2022). At higher temperatures (> 600 °C), steam promotes two opposing processes: (i) enhanced aromatic ring condensation through selective removal of aliphatic and amorphous carbon domains, which increases stability; and (ii) introduction of oxygen-containing functional groups (–OH, –COOH) and creation of disordered carbon structures, which may decrease stability. The net effect on carbon persistence depends on which process dominates, which in turn depends on steam partial pressure, exposure time, and feedstock mineral content (minerals catalyse gasification). Resolving this question will require dedicated aging or incubation studies comparing steam-pyrolysed biochars with controls produced under N_2_ at identical temperatures.

The removal of C from the char surfaces by steam reduces biochar yields but can also promote the formation of total pore volume and micro-sized pores, decreasing the overall pore size (e.g., from 150 µm to 100 µm using 0.15 ml H_2_O per min with 60 g coal at 500 °C; (Chang et al. 2024)) creating a char with a more porous structure and higher surface area (Minkova et al. 2001). Examples of surface area and pore volume/sizes of biochars produced under a steam atmosphere are shown in Table 4. The removal of tar deposits frees up active sites and pores on the char that would otherwise reduce its adsorption capacity (Gilbert et al. 2009; Wang et al. 2021). During biomass pyrolysis, steam promotes devolatilization and prevents the solid–liquid transition of simple sugars and extractives that could block nanopores in biochar (Gargiulo et al. 2018), resulting in micropores (Mandal et al. 2017).

**Table 4:** BET surface area, total pore volume and average pore size of biochar produced under a steam atmosphere

Feedstock	Steam: biomass ratio	Temperature (°C)	BET surface area (m^2^ g^−1^)	Total pore volume (cm^3^ g^−1^)	Average pore size (nm)	Reference
Digested food waste	3 g: 1 g	850	3.97	7,000	7.3	Zhang et al. (2021)
Dermal tissue of oil palm frond	1.5 L: 10 kg	500	422	0.337	10.2	Lawal et al. (2021)
Pinewood sawdust	3 mL: 0.75 g	500	16.5		38	Fernandez et al. (2022)
		600	72.9		4	
Oil palm fronds	12 L: 0.2 kg	600	461.3	0.316	9.4	Lawal et al. (2020a)
Fresh oil palm	12 L: 0.2 kg	500	458	0.316	9.4	Lawal et al. (2020b)
Coconut husk	2 L: 0.5 g	800	189	0.0902	1.7	Fu et al. (2020)
Coal	0.15 mL: 60 g	500			102,670	Chang et al. (2024)
Cellulose fibres P. nigra wood	0.5 g: 6 g	530 560 700 480 600	351 473 593 6 217	0.177 0.799 0.250 0.018 0.121		Gargiulo et al. (2018)

### Summary and conclusion—steam atmosphere

The reduction stage using steam serves as an activation step to increase the specific surface area and micropore volume of the biochar. These are essential properties affecting biochar performance in a range of applications, such as contaminant removal (Lawal et al. 2020a). However, this improvement in the surface properties is at the cost of lower biochar yield and subsequently lower C sequestration potential. Therefore, the pyrolysis of biomass under steam is a promising method when focusing on the production of liquid fuels (Cheng et al. 2017; Fumoto et al. 2011; Hongfu et al. 2002; Kang et al. 2020; Önal et al. 2011) and valuable chemicals (Fumoto et al. 2011; Sonoyama et al. 2011) with some benefits for biochar also (Kantarelis et al. 2014; Mellin et al. 2014; Staš et al. 2014). The presence of steam in the pyrolysis atmosphere favors the formation of high-yield liquid products, while the almost simultaneous pyrolysis and gasification of the fuel result in the formation of solid products with a high surface area and well-developed porous structure.

The development of a highly porous structure and the presence of abundant surface functional groups in steam-derived biochar significantly enhance its performance as an adsorbent in environmental remediation, particularly for wastewater treatment and removal of emerging contaminants (Barszcz et al. 2024). The increased surface area and microporosity also make steam-activated biochar a promising material for catalytic applications and as a precursor for activated carbon in energy storage devices. However, the trade-off between lower yield and potential reductions in carbon sequestration suggests that steam pyrolysis is best suited to scenarios prioritizing high-performance adsorbents and catalysts over maximum carbon retention. Because reported effects on fixed carbon, elemental ratios, and structural indicators do not all move in the same direction, the persistence implications of steam-assisted pyrolysis are treated comparatively in Sect. 11.3.

## Biochar production under oxidative atmosphere

During oxidative pyrolysis, i.e., in an atmosphere containing sub-stoichiometric amounts of oxygen, some of the volatiles released during pyrolysis are oxidized, reducing the yield of liquid products and affecting their composition. The oxidation of pyrolysis products, often through partial combustion, is exothermic. The energy released from the oxidation process can be used internally within the process to drive the endothermic pyrolysis reactions. Key reactions and expected product/char trends are summarized in SI Table S1.

The process energy balance is very sensitive to O_2_ concentration in the pyrolysis atmosphere, as shown when the O_2_ concentration was gradually increased. Under an inert and 1% O_2_ atmosphere, the primary breakdown of cellulose was clearly endothermic, and the endothermic peak shrank as the O_2_ content increased (Zhao et al. 2015). Therefore, the energy needed for the primary thermal degradation of biomass and the subsequent secondary reactions can be partially provided by in situ heating. Oxidative pyrolysis has the potential to become an autothermal technology that can be self-sustaining through introducing the optimum amount of oxygen that can be determined based on the enthalpy of the pyrolysis of the specific biomass (Huang et al. 2020).

### Yield and composition of pyrolysis liquids and gases

Pyrolysis under an oxidative atmosphere can promote the yields of water and permanent gases, mainly CO_2_, and CO as a result of heterogeneous oxidation reactions and secondary reactions of nascent volatiles (Zhao et al. 2019, 2014). This reduces the heating value of the gaseous products compared to pyrolysis under an inert gas atmosphere that forms more energy-rich gases, such as CH_4_ (Zhao et al. 2019, 2014). Furthermore, oxidative pyrolysis atmospheres decrease the yield of pyrolysis liquids and affect their composition (Daouk et al. 2017; Zhao et al. 2019, 2014).

Although published research on this topic is limited, some important trends have been reported in the available literature. Oxidative pyrolysis can accelerate the conversion of primary tar to secondary and tertiary tar via an in situ exothermic process and active radical attack (Zhao et al. 2019). Furthermore, small quantities of O_2_ (0.525–1.05% v/v) in the purge gas can boost the yield of hydrolysable sugars and the total yield of phenolic monomers (Kim et al. 2014), highlighting that adding some O_2_ during pyrolysis could increase the content of high-value chemicals, such as phenolic compounds (Kim et al. 2014).

### Yield and properties of biochar

The yield of biochar decreased considerably with the increase in O_2_ concentration in the pyrolysis atmosphere by up to 50% in high-temperature pyrolysis (700 °C) (Zhao et al. 2019, 2015). The biochar yield of rice straw, for example, decreased from 35.4 to 15.1 wt% during pyrolysis at 800 °C with the O_2_ concentration increasing from 0 to 21% (i.e., air but supplied in a sub-stoichiometric amount) (Zhao et al. 2015). Similarly, for low-temperature pyrolysis (300 °C), the biochar yield decreased from 76 to 51 wt%, highlighting that the O_2_ partially reacted with the biomass already at such low temperatures (Zhao et al. 2019). This increases exothermic reactions, decreasing the energy input requirements, with potential for an autothermal process with pyrolysis gases/liquid recovery or higher energy outputs when pyrolysis gases are burnt (Brown et al. 2024; Cavalloni et al. 2025; Milhé et al. 2013).

Biochar’s carbon stability might also be influenced by the presence of O_2_ in the pyrolysis atmosphere. Heterogeneous oxidation at temperatures >400 °C can lead to the breakdown of weak bonds, aiding the formation of aromatic rings (Zhao et al. 2015). This results in a biochar with a higher degree of aromaticity (as shown in NMR and FTIR studies) (Lu et al. 2025; Park et al. 2019), lower O/C and H/C ratios, both indicators of higher biochar stability, and in some cases also improved thermal stability (Gao et al. 2024c, 2024a, 2024b). Such biochar could be more stable in the environment, i.e., recalcitrant to degradation, resulting in slower re-release of C into the atmosphere. However, wildfire charcoals have also been shown to exhibit lower aromaticity and higher H/C and O/C ratios compared to biochars produced under controlled conditions, primarily a result of the presence of O_2_ in the environment and shorter residence times during forest fires (Santín et al. 2017). Therefore, the stability of biochar produced under low concentrations of O_2_ could be important to investigate, specifically under field settings.

While the decrease in biochar yield due to oxidation may be undesirable, the properties of biochar can be improved during oxidative pyrolysis, especially physical and chemical properties, partially compensating for the lower yields. Previous studies found a considerable increase in surface area of biochar from oxidative pyrolysis compared to pyrolysis under an inert atmosphere, such as an increase in the BET surface area of *Phragmites australis* biochar produced at 675 °C from 307 to 350 m^2^ g^−1^ with the addition of 9% oxygen to an N_2_ atmosphere (rotary tube furnace) (Jiang et al. 2021). With the addition of 21% O_2_ to the pyrolysis environment, the BET surface area of pinewood biochar (produced in a bench-scale fixed-bed gasifier) increased from negligible (~ 2) to 75 m^2^ g^−1^ and ~ 375 m^2^ g^−1^ when the biochars were produced at 400 and 500 °C, respectively (Zhao et al. 2014). However, when the same biochar was produced at 600 °C, the addition of oxygen only marginally increased the surface area, and when produced at 700 °C, even a slight decline in surface area is visible (Zhao et al. 2014). This highlights the temperature–oxygen concentration interactions during biomass pyrolysis that need to be considered to optimize outcomes.

Pyrolysis in an oxidative atmosphere also enhances the formation of micropores and inhibits biochar thermal annealing and ordering of carbon structure at high temperature (Yip et al. 2011), yielding biochar with higher porosity (Lu et al. 2025; Zhao et al. 2019). Such changes increased the biochars' sorption capacity as demonstrated for phenol sorption (Gao et al. 2024a). Oxidative atmospheres also produced biochar with a higher content of acidic surface functional groups, including carboxyl and hydroxyl groups and a non-protonated aromatic C fraction that increases surface negative charges and hence biochar’s cation exchange capacity (Lu et al. 2025; Park et al. 2019; Suliman et al. 2016).

The pronounced changes in biochar properties under oxidative pyrolysis arise from heterogeneous reactions between substoichiometric O_2_ and the carbon matrix. At temperatures > 300 °C, O_2_ initiates partial combustion, cleaving weaker C–C and C–H bonds in aliphatic and amorphous carbon domains. This promotes the formation of oxygen-containing surface groups (carboxyl, hydroxyl, carbonyl) via electrophilic addition of O_2_-derived radicals (O•, OH•) to aromatic rings. These reactions increase surface acidity and inhibit carbon structure annealing, preserving micropores and creating disordered char (Gao et al. 2024b).

Inorganic constituents, particularly potassium in biomass ash, act as catalysts by lowering activation energies for oxidation. Thermogravimetric and fluidized bed studies demonstrate that potassium enhances CO/CO_2_ formation rates through oxygen shuttling mechanisms, accelerating carbon oxidation (Peterson and Brown 2020). Concurrently, oxidative attrition generates fine biochar particles with higher surface reactivity, further amplifying adsorption capacity and cation exchange.

### Summary and conclusion—O_2_ atmosphere

Adding O_2_ during pyrolysis substantially changes the process kinetics of biomass conversion, producing more energy output and more permanent gases (Brown et al. 2024; Cavalloni et al. 2025; Milhé et al. 2013). It also changes the pyrolysis liquid formation, which could be beneficial for the production of high-value chemicals (Kim et al. 2014). The biochar yield can drop substantially with O_2_ addition. However, the pronounced increase in surface area, microporosity, and acidic surface functional groups resulting from oxidative pyrolysis atmospheres imparts biochar with enhanced cation exchange capacity and reactivity. These features are advantageous for use as soil conditioners to improve nutrient retention and as functional materials for pollutant adsorption. The potential for increased aromaticity and environmental stability may also benefit long-term soil carbon storage, though further research is needed to better understand increased carbon loss, chemical/thermal carbon stability and subsequent biochar carbon stability, under field conditions.

Any apparent increase in aromaticity or improvement in stability proxies under mild oxidative conditions should be weighed against the often substantial decrease in biochar yield; this trade-off is synthesized in Sect. 11.3.

## Biochar production under NH_3_ atmosphere

A large number of studies have shown a positive effect of N atoms in biochar for performance in a range of applications, such as environmental remediation or energy storage (Leng et al. 2020). Different methods can be used to obtain N-enriched biochar, including the use of N-rich feedstock, chemical additives, and a pyrolysis atmosphere rich in reactive N. However, simple pyrolysis under a N_2_ atmosphere has a negligible effect on N content in biochar and is therefore not a viable way of introducing N functional groups to biochar, as molecular N is relatively unreactive (Liu et al. 2018a, b). On the other hand, ammonia (NH_3_) in the pyrolysis atmosphere can be very effective in increasing the N content of biochar, especially at temperatures below 400 ℃, as above this temperature NH_3_ starts decomposing. Key reactions and expected product/char trends are summarized in SI Table S1.

**Fig. 3:**
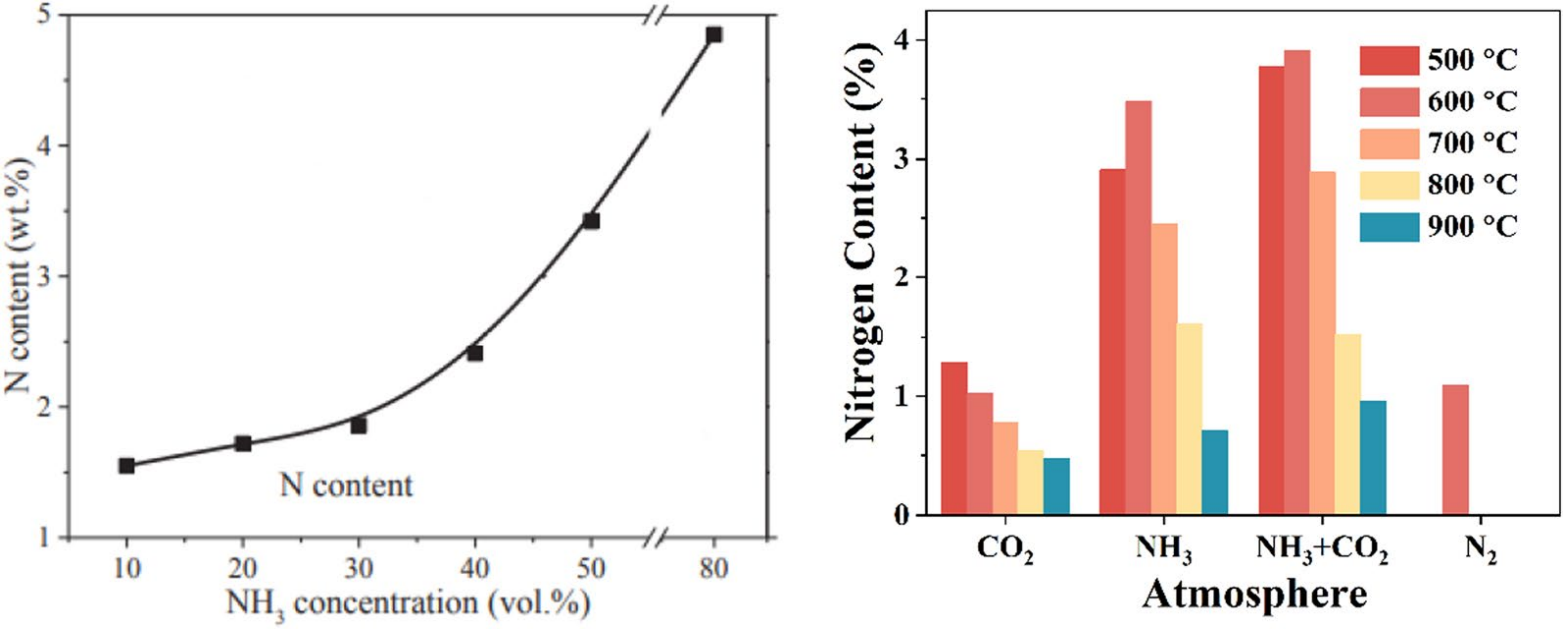
**a** Nitrogen content in biochar produced by pyrolysis under an NH_3_ atmosphere (Wei Chen et al. 2018a, b); **b** Nitrogen content variation of biochar under different atmospheres (Zhang et al. 2014)

Biomass pyrolysis in a specific NH_3_-rich atmosphere, namely thermo-catalytic conversion and ammonization (TCC–A), has been developed recently, with the primary aim to introduce N functional groups to biochar or to produce speciality chemicals. Research on NH_3_/N low-temperature pyrolysis pretreatment of biomass showed that pyrolysis under NH_3_ was more effective in enhancing N fixation and reducing the release of N-containing volatiles in biochar compared to pyrolysis under N_2_. This resulted in a much higher N-doping ratio in biochar prepared under an NH_3_ atmosphere (Zheng et al. 2022). Doping ratios as high as 3.82%, 7.00%, and 8.10% have been reported when low-temperature pyrolysis under NH_3_ was used (Guo et al. 2016; Li et al. 2019; Zhao et al. 2022). Such atmospheres typically consist of pure NH_3_, N_2_ + NH_3_, or Ar + NH_3_ with varied concentrations of NH_3_ (W. Chen et al. 2018a, b; Leng et al. 2020; Liu and Huang 2018). The concentration of NH_3_ in the pyrolysis atmosphere is a key parameter that influences the conversion behaviors of the biomass and the properties of the resulting biochar. Recent studies have shown that N content in biochar increases considerably with higher concentrations of NH_3_ in the pyrolysis atmosphere, as shown in Fig. 3.

### Yield and properties of biochar

Pyrolysis in an NH_3_ atmosphere yields biochar with a range of N-functional groups, with improved performance in oxygen reduction, catalysis, pseudo-capacitance, and waste liquids treatment (Guo et al. 2016; Li et al. 2019; Shen and Fan 2013). XPS analysis indicated an increase of N content from 0.3–4%, clearly highlighting the potential for incorporation of N into the biochar structure due to the NH_3_ atmosphere. During pyrolysis, NH_3_ decomposes at elevated pyrolysis temperatures (> 300°C) into reactive nitrogen species (NH*, NH_2_*, N*) through radical chain reactions (Davidson et al. 1990). These radicals interact with the carbon matrix through substitution and addition pathways, forming stable nitrogen functionalities such as pyridinic-N and pyrrolic-N (Liu et al. 2018a, b; Liu et al. 2020). These radicals etch carbon fragments, enlarging pore structures while simultaneously incorporating nitrogen functionalities (pyridinic-N, pyrrolic-N, quaternary-N) into the biochar. This one-step process combines nitrogen doping with pore development, as NH_3_ acts both as a nitrogen source and a mild activating agent. The enhanced electron density and surface polarity from nitrogen groups improve Lewis acid–base interactions and chemisorption capacity, critical for pollutant removal and catalytic applications (Su et al. 2022; Tang et al. 2024). A characterisation of N-functional groups in bamboo biochar produced by NH_3_ pyrolysis clearly showed four typical N-containing functional groups (Li et al. 2019), specifically pyridinic-N (398.5 ± 0.3 eV), pyrrolic-N (400.5 ± 0.3 eV), quaternary-N (401.2 ± 0.3 eV), and oxidized-N (403.2 ± 0.3 eV) (Ma et al. 2019).

Pyrolysis in an NH_3_ atmosphere did not affect biochar yield compared to an inert atmosphere (Chen et al. 2016). However, NH_3_ increased the BET surface area substantially from 158 to 521 m^2^ g^−1^ and the total pore volume from 0.075 to 0.248 cm^3^ g^−1^. Combining chemical pretreatment of biomass via K_2_C_2_O_4_ and KHCO_3_ with pyrolysis in an NH_3_ atmosphere increased the surface functionality of biochar substantially compared to pretreatment only (Li et al. 2021). This, subsequently, improved biochar’s performance in the removal of phenol from wastewater simulated using designed phenol solutions, achieving a removal rate of 170 mg g^−1^ compared to ~105 mg g^−1^ for biochar without N-doping. This improved performance was mainly due to improved physical structure, such as the formation of internal pore volume including micropores, which further increased its surface area and chemical properties, such as enhanced Lewis acid–base interaction between pyridinic and pyrrolic-N and phenols, as well as high electronegativity of N, and chemisorption (Inyang and Dickenson 2015; Lian et al. 2016).

### Summary and conclusion—NH_3_ atmosphere

The high nitrogen content and the formation of pyridinic, pyrrolic, and quaternary nitrogen groups in NH_3_-derived biochar markedly improve its performance in environmental remediation, especially for the adsorption of organic pollutants and heavy metals in wastewater (Dong et al. 2019; Kang et al. 2019; Mian et al. 2019). Additionally, the enhanced surface area and tailored nitrogen functionalities support applications in catalysis and electrochemical energy storage, such as supercapacitor electrodes. These application-specific benefits highlight the value of NH_3_-assisted pyrolysis for producing engineered biochar with targeted functionalities.

## Biochar production under flue-/pyrolysis gas atmosphere

Flue gas from the combustion of gaseous/liquid pyrolysis products is readily available on the site of industrial pyrolysis processes for biochar production. It can be advantageously used as a carrier or purge gas. The main components of flue gas are N_2_, CO_2_, and steam, as well as smaller amounts of other gases, including O_2_. Pyrolysis of biomass in a flue gas atmosphere, therefore, brings together aspects discussed in different parts of this paper. Key reactions and expected product/char trends are summarized in SI Table S1. Surprisingly, although flue gas has been used extensively as a carrier gas in industrial applications for a long time, there is only minimal research on the effects of flue gas composition and comparison against other atmospheres in the available literature.

Among the different technologies for biochar production in a flue gas atmosphere, the Lambiotte retort is a good example of a large-scale industrial unit using this approach. The hot flue gas from the combustion of the pyrolysis gases is used directly as a heating agent in a moving bed of biomass and biochar. This technology produces biochar with a relatively high yield (30–35%) and a high fixed carbon content (80–90%) (Morello 2015). Another example is the multiple hearth reactor, where hot flue gas from the combustion of pyrolysis gases is directly introduced at the bottom of the reactor and flows against the flow of biomass and biochar, heating the material at the different levels in the reactor and carrying away produced volatiles (Vallero 2014).

From a mechanistic perspective, the effects of flue-/pyrolysis gas atmospheres on biochar formation represent a superposition of the individual gas-phase interactions discussed in earlier sections. The CO_2_ component drives Boudouard-type etching of the carbon matrix, the steam fraction promotes water–gas reactions and pore development, and any residual O_2_ initiates partial oxidation, collectively accelerating devolatilization and reducing char yield relative to pure N_2_. The net effect on biochar properties depends critically on the relative concentrations of these reactive species, which in turn vary with combustion efficiency, fuel type, and flue-gas recirculation rate. This compositional variability makes it difficult to compare results across studies that use different simulated gas mixtures, and underscores the need for standardized reporting of gas compositions in future work.

The direct integration of combustion of pyrolysis gases and possibly vapors offers an easy and efficient way of heating the pyrolysis process. This eliminates or at least reduces the need for external fuel/heat supply to sustain the pyrolysis reaction, thus improving the process's economics.

### Yield and properties of biochar

There can be issues with the adoption of this approach for biochar production related to contaminants, especially when used for the production of biochar destined for use in agriculture or environmental management, where organic and inorganic pollutants need to be minimized (Uday et al. 2022). The presence of contaminants in flue gases, such as hydrogen chloride and hydrogen fluoride, as well as hydrocarbon derivatives and heavy metal derivatives, has been reported, and their concentrations depend on the fuel used and the combustion process (Villeneuve et al. 2012). Therefore, flue gas pyrolysis atmospheres need to be thoroughly investigated for use in biochar production, taking into consideration the different requirements of biochar applications. At present, there is insufficient published research on this topic, mostly focused on low-temperature pyrolysis and torrefaction.

The response of biochar yield and stability is substantially affected by the composition of the flue gas atmosphere. A simulated pyrolysis gas atmosphere containing 20% CH_4_, 10% CO_2_, and 70% N_2_ added during pyrolysis of municipal solid waste (600 and 800 °C) reduced biochar yields marginally by ~ 2% compared to N_2_ only, but slightly increased the fixed C content by a similar margin and decreased H/C and O/C ratios, also indicators of higher carbon stability (Yan et al. 2020; Crombie et al. 2013). The presence of CO_2_ and CH_4_ in the flue gas facilitated devolatilization reactions, such as the associated Boudouard reaction (CO_2_ + C-biochar → CO), lowering the biochar yield (Yan et al. 2020). Simulated pyrolysis gas atmosphere mainly containing CO_2_, CO, and CH_4_ marginally decreased the yield and fixed C content of biochar in comparison with N_2_ atmosphere during low-temperature pyrolysis at 300 °C (torrefaction) (Xu et al. 2019). Torrefying/pyrolyzing spruce wood at 275 °C in an atmosphere with steam and CO_2_ also did not majorly affect biochar yield; however, adding 10% O_2_ into the pyrolysis atmosphere considerably decreased biochar yield from 83.1% to 76.4% (Tran et al. 2016). Oxygen addition, however, did not affect the fixed C content during pyrolysis at this lowest end of the temperature spectrum. There is also some evidence for an increased BET surface area using a pyrolysis gas atmosphere mainly containing CO_2_, CO, and CH_4_ compared to a N_2_ atmosphere. However, this was only tested during torrefaction at 260 °C (Xu et al. 2019).

### Summary and conclusion: flue-/pyrolysis gas

Current evidence is too limited and composition-dependent to support a general conclusion on carbon retention or long-term persistence under flue-/pyrolysis gas atmospheres. Some studies suggest that selected mixed-gas conditions can produce biochars with relatively high fixed carbon content and moderate surface area, but these findings remain difficult to generalize across systems. The potential presence of contaminants necessitates careful assessment before use in agricultural or environmental remediation contexts. The integration of flue gas recycling can also enhance process economics and sustainability in large-scale biochar production. The composition of pyrolysis flue-/pyrolysis gas varies substantially, and hence, the few studies that tested such atmospheres are difficult to compare. The O_2_ concentration clearly has the greatest effect on pyrolysis process kinetics, including biochar yield (Bach et al. 2017; Tran et al. 2016). More studies testing the effects of simulated and actual flue-/pyrolysis gas atmospheres are urgently required, given the clear advantages of recycling pyrolysis gases for the economics of biochar production units. Because flue-/pyrolysis gas atmospheres are mixtures whose composition varies substantially across systems, current evidence is insufficient to support a general conclusion on carbon retention or long-term persistence; this limitation is reflected in the comparative synthesis in Sect. 11.

## Biochar production in a CH_4_ atmosphere

Methane is a weakly reducing gas that, under pyrolysis conditions, can act both as a hydrogen donor (via thermal cracking above ~700 °C) and as a carbon source through catalytic methane decomposition (CMD) on the biochar surface. Key reactions and expected product/char trends are summarized in SI Table S1. Despite the practical relevance of CH_4_—it is a major component of pyrolysis gas and biogas, and thus readily available for gas recirculation in industrial pyrolysis systems—surprisingly, little research has been conducted on its effects on biochar properties.

During fast pyrolysis of corncobs in a fluidized-bed reactor at 500 °C, CH_4_ did not significantly affect biochar yield compared to N_2_ (Zhang et al. 2011). Similarly, torrefaction of camellia shell at 260 °C under CH_4_ marginally increased BET surface area (from 1.16 to 1.29 m^2^ g^−1^), a difference likely within analytical uncertainty, without affecting yield (Xu et al. 2019). However, Xu et al. (2020) pyrolyzed pine wood at 500 °C in a fixed-bed reactor under N_2_, H_2_, CO_2_, CH_4_, and CO_2_/CH_4_ mixtures and found that CH_4_ gave the highest overall biomass conversion (76.9 wt%, approximately 2% higher than N_2_) (Xu et al. 2020). The CO_2_/CH_4_ mixture promoted bio-oil yield by approximately 1% and shifted bio-oil composition toward alcohols, aldehydes, ketones, and furans at the expense of phenols and sugars, consistent with simultaneous methane bi-reforming with CO_2_ and steam generated during pyrolysis (Xu et al. 2020). The same study also observed distinct gas product distributions under CH_4_, with increased CO and H_2_ yields indicative of methane reforming reactions occurring alongside biomass devolatilization.

At higher temperatures, CH_4_ interacts with the biochar surface through catalytic methane decomposition, depositing solid carbon. Patel et al. (2020) demonstrated that biochar and activated char from biosolids pyrolysis achieved maximum CH_4_ conversions of 65.2% and 71.0%, respectively, at 900 °C with 10% CH_4_ in N_2_ (Patel et al. 2020). Temperature strongly controlled the morphology of deposited carbon: carbon nanofibers formed at 700 °C, while nanospheres formed at 900 °C. Harun et al. (2020) reported that Douglas fir biochar with exceptionally high surface area (3256 m^2^ g^−1^, 82% microporous) maintained approximately 51% methane conversion over 60 h at 800 °C, with carbon nanotube growth observed on the biochar surface (Harun et al. 2020). Ash components, particularly K_2_O, Na_2_O, CaO, and MgO, provided strong metal–support interactions enhancing catalytic activity and carbon deposition. More recently, in situ graphitic carbon deposition from methane decomposition on balsa wood biochar (700–1000 °C) has been shown to create conductive networks with enhanced graphitization (Cui et al. 2025), suggesting that CH_4_ at elevated temperatures can fundamentally modify the carbon microstructure and electrical properties of biochar.

These findings from CMD studies, while conducted primarily with pre-formed biochars as catalytic substrates rather than during pyrolysis itself, suggest that when CH_4_ is present in the pyrolysis atmosphere at temperatures above ~ 700 °C, carbon deposition on the nascent char surface may occur in situ, potentially increasing char yield and modifying surface morphology and electrical conductivity. The practical relevance of this mechanism for pyrolysis gas recycling, where CH_4_ concentrations typically range from 5–30 vol%, warrants systematic investigation.

Very little research has been undertaken on the slow pyrolysis of biomass in a CH_4_ atmosphere in the typical biochar-oriented temperature range (350–750 °C) with comprehensive biochar characterization. The interaction between CH_4_ and CO_2_ as co-components of biogas or pyrolysis gas is of particular interest, as their opposing reductive and oxidative characters may produce synergistic effects on biochar properties that differ from either gas alone (Lee et al. 2021). More targeted studies examining the effects of CH_4_ and CH_4_-containing gas mixtures on biochar yield, porosity, surface chemistry, carbon stability, and functional performance are urgently needed.

## Comparative assessment of pyrolysis atmospheres

The results reviewed in the preceding sections show that the pyrolysis atmosphere strongly influences both the distribution of pyrolysis products and the properties of the resulting biochar. Inert atmospheres, typically N_2_ or Ar, provide the baseline against which the effects of more reactive gases can be understood. By comparison, atmospheres containing CO_2_, steam, O_2_, NH_3_, or components of flue-/pyrolysis gas can alter biomass decomposition pathways, promote secondary gas–solid reactions, and modify the chemical and structural development of the carbon matrix. These changes can be beneficial where the aim is to tailor biochar properties or shift the balance of products towards gases or liquids, but they may also reduce biochar yield or complicate the interpretation of carbon stability. The choice of atmosphere should therefore be considered in relation to the intended application of the biochar and the wider objectives of the pyrolysis process.

### Comparative effects on product distribution

Under inert atmospheres, pyrolysis generally follows the most conservative pathway, with comparatively high retention of carbon in the solid fraction and without additional reactions between the atmosphere and the evolving pyrolysis products. This makes N_2_ and Ar suitable reference atmospheres for studies where biochar yield is a primary concern and where changes in product composition are intended to arise mainly from feedstock type and thermal conditions rather than from interactions with the carrier gas.

More reactive atmospheres shift the product distribution away from this baseline in different ways. CO_2_ most consistently increases gas production, particularly CO, while reducing tar/oil yield, especially at higher temperatures where secondary reactions become more pronounced. Steam can also alter the balance of products substantially, often favoring greater formation of condensable liquids or modifying their composition, while at the same time lowering biochar yield. Oxidative atmospheres containing low concentrations of O_2_ have the strongest effect on product yields, as oxidation reactions accelerate biomass decomposition and promote the conversion of solid and condensable products into gases. This can be advantageous where the process is designed to increase gas output or reduce tar formation, but it is clearly disadvantageous where the main objective is to maximize biochar production.

The effects of NH_3_, CH_4_, and flue-/pyrolysis gas atmospheres are less clearly established. NH_3_ has mainly been studied for its influence on biochar chemistry rather than for systematic changes in product distribution. CH_4_ remains particularly underexplored, and the few available studies do not yet support a clear conclusion regarding its effect on biochar yield under typical pyrolysis conditions. Flue-/pyrolysis gas atmospheres are especially difficult to generalize because they are mixtures whose composition can vary substantially, meaning that the relative importance of CO_2_-, steam-, and O_2_-driven reactions differs between systems. For this reason, apparent inconsistencies in product yields under such atmospheres are not surprising and should not be interpreted as directly contradictory without close attention to gas composition and process severity.

Overall, the available evidence indicates that inert atmospheres remain preferable when the main objective is high biochar yield, whereas reactive atmospheres become more attractive when the process is intended to suppress tar formation, increase gas production, or provide additional process benefits such as heat integration or autothermal operation.

### Comparative effects on biochar functionality

A clear advantage of reactive atmospheres is their capacity to modify biochar properties during pyrolysis itself, reducing the need for separate post-treatment or activation steps. In comparison with inert conditions, atmospheres containing CO_2_, steam, O_2_, and NH_3_ often produce biochars with greater specific surface area, higher pore volume, and more developed surface chemistry. These changes are generally attributed to enhanced cracking of volatile compounds, reactions between the gas atmosphere and the carbon matrix, and the formation or exposure of surface functional groups.

Among the atmospheres considered, CO_2_ and steam show the most consistent evidence of increased surface area and pore development, particularly as the pyrolysis temperature increases. In both cases, the atmosphere can promote reactions that remove less-ordered carbon and open the pore structure, thereby improving the accessibility of the internal surface. Oxidative atmospheres can have a similar effect, but they do so through a more aggressive pathway and often with a greater penalty in biochar yield. NH_3_ is distinct in that its main effect is not only structural but also chemical, as it can introduce nitrogen-containing surface functionalities that are of particular interest for sorption and catalytic applications.

These modifications are important because they can enhance biochar performance in applications where surface reactivity and pore architecture are critical, such as contaminant sorption, catalysis, nutrient retention, or electrochemical uses. At the same time, improved functionality should not be viewed in isolation. The same conditions that increase porosity or functionalization often also intensify carbon loss from the solid phase. A more functionalization biochar is therefore not necessarily the most suitable product when the main purpose of pyrolysis is carbon sequestration.

The evidence for functional changes under flue-/pyrolysis gas and CH_4_ atmospheres is currently limited. Some studies indicate that mixed pyrolysis gas atmospheres may modestly affect surface area or fixed carbon content, but the effects are difficult to separate from the influence of the individual gas components. For CH_4_, the reported differences in surface area are very small and may fall within analytical uncertainty. As a result, the literature currently supports a strong conclusion for the ability of several reactive atmospheres to enhance biochar functionality, but only a weak conclusion for CH_4_ and a highly conditional one for flue-/pyrolysis gas.

### Carbon retention and persistence across atmospheres

The relationship between pyrolysis atmosphere and carbon sequestration potential is more complex than the effects on surface area or product distribution alone. In this context, it is important to distinguish between carbon retention, stability proxies, and long-term environmental persistence. Carbon retention refers to the proportion of feedstock carbon remaining in the solid product after pyrolysis. Stability proxies include properties such as fixed carbon content, H/C and O/C ratios, aromaticity, and thermal stability, which are often used as indicators of recalcitrance. Environmental persistence refers more directly to the resistance of the retained carbon to decomposition after application. These parameters are related, but they are not interchangeable, and they do not always change in the same direction.

From the perspective of retained solid carbon, inert atmospheres remain the most reliable option because they avoid the additional gasification, oxidation, or activation-like reactions that reduce biochar and carbon yield under more reactive conditions. This is one reason why inert pyrolysis continues to be the most straightforward route where biochar is produced primarily for long-term carbon storage. However, a high biochar and carbon yield alone does not guarantee persistence, and therefore both the quantity and the nature of the retained carbon must be considered.

CO_2_ illustrates this balance particularly well. At moderate pyrolysis temperature, CO_2_ can in some cases favor secondary char formation or preserve fixed carbon, but at higher temperatures it also promotes gas–solid reactions that reduce biochar yield. The available studies suggest that the effect depends strongly on feedstock type, ash content, biomass form, and process conditions. Similarly, some studies report increases in aromaticity or thermal stability under CO_2_ atmospheres, while others show reduced carbon retention at higher temperature. CO_2_ should therefore not be regarded as either uniformly favorable or uniformly unfavorable for carbon sequestration; rather, its effect depends on the balance between structural development of the retained carbon and the extent of carbon loss from the solid phase.

Steam presents a related but somewhat different ambiguity. It often enhances pore development and can modify surface oxygen functionalities, but its effect on stability is less straightforward. Some reported changes, such as increased aromatic condensation under certain conditions, may suggest greater recalcitrance, whereas others, including reduced fixed carbon or increased structural disorder, do not point in the same direction. For this reason, the effect of steam on long-term carbon persistence cannot yet be generalized with confidence. What can be said more clearly is that steam tends to favor functional development of the biochar at the expense of higher solid yield.

Oxidative atmospheres also need to be interpreted with care. Mild oxidation can reduce H/C and O/C ratios and may promote formation of a more condensed carbon structure under controlled conditions. At the same time, O_2_ leads to the greatest loss of biochar yield among the atmospheres considered here. As a result, improved values of some stability proxies do not necessarily translate into superior overall carbon retention. In addition, comparison with wildfire-derived chars should be made cautiously, since wildfire charring and controlled oxidative pyrolysis represent different thermal and oxidative histories.

For NH_3_, CH_4_, and flue-/pyrolysis gas atmospheres, the available evidence is not yet sufficient to support firm conclusions on carbon persistence. Most NH_3_ studies focus on N-doping and functional properties rather than on stability. CH_4_ has been investigated only rarely under typical pyrolysis conditions. Flue-/pyrolysis gas atmospheres are particularly difficult to assess because their composition varies and because potential contaminant issues must also be considered when judging the suitability of the resulting biochar for environmental applications.

Taken together, the evidence indicates that atmospheres which most strongly improve biochar functionality are not necessarily those that maximize retained persistent carbon. Where carbon sequestration is the dominant objective, the relevant question is not only whether a reactive atmosphere lowers the H/C or increases aromaticity, but also whether sufficient carbon is retained in the solid phase and whether the available stability indicators provide convincing support for long-term persistence. By contrast, where the aim is adsorption, catalysis, or other performance-driven applications, lower-yield but more strongly modified biochars may still be entirely appropriate. This distinction should be reflected more clearly in future studies, which should report both yield and stability-related parameters in a way that allows these trade-offs to be evaluated directly.

### Evidence maturity and research priorities

The available evidence is uneven across the atmospheres considered in this review. CO_2_, steam, and O_2_ have been studied sufficiently to support broader comparison, although important contradictions remain and are often linked to differences in feedstock, temperature range, gas concentration, and reactor configuration. NH_3_ is less widely studied, but the literature already points to a clear niche in the one-step production of nitrogen-functionalized biochars. By contrast, CH_4_ and flue-/pyrolysis gas atmospheres remain underexplored, and conclusions about their effects on biochar properties should therefore be drawn with caution.

A key priority for future work is not simply to increase the number of studies, but to improve comparability between them. More systematic investigations are needed in which atmospheres are compared under matched conditions and where the feedstock properties, process parameters, and gas compositions are reported in sufficient detail. This is particularly important for resolving the apparently contradictory findings identified in the literature, many of which are likely to reflect differences in process severity or in the boundary between pyrolysis and incipient gasification rather than true disagreement.

Future studies should also place greater emphasis on the relationship between biochar/carbon yield and long-term carbon stability. At present, many studies report changes in surface area, elemental composition, or aromaticity without linking these changes to overall retained carbon or to direct evidence of environmental persistence. This makes it difficult to judge whether a given atmosphere is advantageous for carbon sequestration, even where it clearly improves some biochar properties. Better integration of these aspects would significantly strengthen the evidence base.

**Fig. 4:**
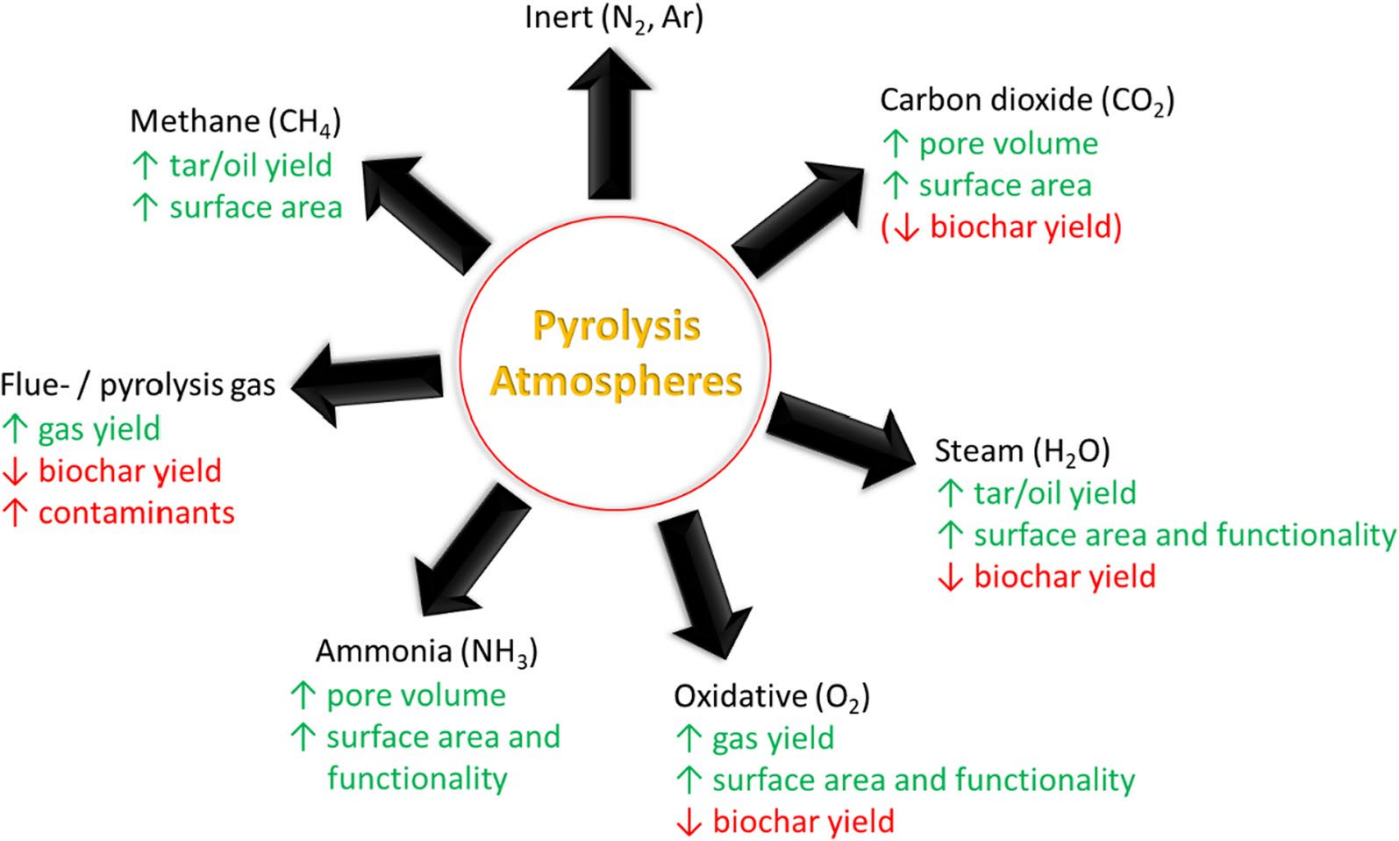
Comparative summary of advantages and disadvantages of different pyrolysis atmospheres on pyrolysis product yields and biochar quality

**Fig. 5:**
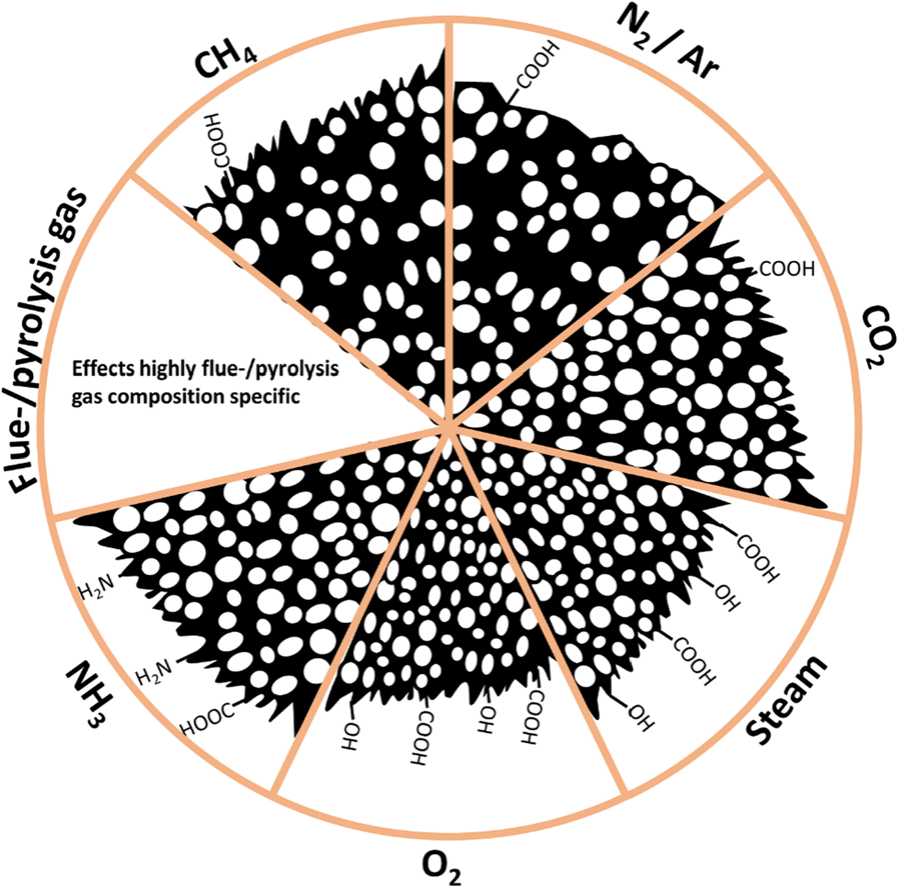
Effect of pyrolysis atmospheres on surface properties (surface area and functionality), pore space and biochar yield (assuming that all other pyrolysis conditions remain the same)

In summary, the pyrolysis atmosphere should be selected according to the intended balance between product yields, biochar functionality, and carbon retention and stability. Inert atmospheres remain the most appropriate benchmark where high biochar yield and conservative carbon retention are prioritized. Reactive atmospheres, particularly CO_2_, steam, O_2_, and NH_3_, offer clear opportunities for engineering biochar properties and altering product distributions, but these benefits usually come with trade-offs in biochar yield, process complexity, or interpretive certainty. A more systematic treatment of these trade-offs is essential if pyrolysis atmospheres are to be used deliberately to produce biochar for specific environmental, agricultural, or industrial applications (Figs. 4, 5, Table 5).

**Table 5:** Comparative summary of pyrolysis atmospheres on pyrolysis fraction yields and biochar properties. Notation: + + strong positive, + moderate positive, 0 no significant effect, − moderate negative, − − strong negative, ? contradictory/insufficient evidence, NA no data available. All effects relative to inert (N_2_/Ar) baseline.

Inert (N_2_, Ar)	Baseline	Baseline	Baseline	Baseline	Baseline	Baseline	Reference condition for all comparisons
Biochar yield	− /0^*a*^	−	− −	0	− ^*d*^	0	CO_2_: ~ neutral < 600 °C, negative > 600 °C (Boudouard). O_2_: yield drops with increasing O_2_ conc. at all T. Flue-/pyrolysis gas: depends on O_2_ fraction
Gas yield	+	− ^*b*^	+ +	*NA*	+	*NA*	CO_2_: sharp increase > 450 °C, especially CO. O_2_: permanent gas increases at all T
Tar/oil yield	−	+	−	*NA*	*NA*	+	CO_2_: tar cracking intensifies > 550 °C. Steam: liquid yield generally increases
Specific surface area	+ to + +	+ to + +	+ to + +	+ to + +	**?** ^*e*^	0/NA ^*f*^	CO_2_: pronounced > 600 °C. O_2_: highest increase at 400–500 °C; may decline > 700 °C. NH_3_: increase linked to N-radical etching
Pore volume	+	+	+	+	*NA*	*NA*	CO_2_/steam/O_2_: micropore development increases with T
O/C ratio	**?** ^*g*^	+	−	*NA*	−	*NA*	O_2_: lower O/C at > 400 °C via aromatisation. Steam: O-functional groups increase O/C
Biochar stability /fixed C content	**?** ^*h*^	**?** ^*i*^	**?** ^*j*^	*NA*	0/ + ^*k*^	*NA*	CO_2_: varies by feedstock, pelleting, and T. O_2_: aromaticity increases > 400 °C but field analogues show lower stability. Steam: competing mechanisms unresolved
Surface functional groups	−	+	+	+ (N-groups)	*NA*	*NA*	NH_3_: most effective < 400 °C (NH_3_ decomposes above). O_2_: acidic groups increase at all T
Contaminants	*NA*	*NA*	*NA*	*NA*	** − /?** ^*l*^	*NA*	Flue-/pyrolysis gas: risk of HCl, HF, heavy metals depending on fuel source and combustion quality

^a^CO_2_ effect on biochar yield is temperature-dependent: approximately neutral at 300–600 °C (may slightly increase yield via repolymerisation), negative at >600 °C due to Boudouard gasification.

^b^Steam may reduce total gas yield at moderate T, while water–gas reactions increase H_2_ and CO at higher T.

^d^Flue-/pyrolysis gas effect depends on composition: 10% O_2_ addition substantially reduced yield (Tran et al. 2016); CO_2_+CH_4_ mixtures caused only marginal reduction (Yan et al. 2020).

^e^Limited evidence: BET surface area increased during torrefaction (260 °C) under a CO_2_/CO/CH₄ mixture (Xu et al. 2019). No data at typical pyrolysis temperatures.

^f^CH_4_ effect at torrefaction T was within analytical error (1.16 vs 1.29 m^2^ g^−^^1^; Xu et al. 2019). No data at typical pyrolysis T.

^g^CO_2_ effects on O/C are inconsistent: some studies show decrease, others increase.

^h^Contradictory. Fixed C increased for pepper stalks at 600 °C (J. Lee et al. 2017d), decreased for sewage sludge at 500–700 °C (Kończak et al. 2020), and depended on pelleting for oil palm fiber (Chen & Lin 2016).

^i^Contradictory. Fixed C decreased at 600 °C but was unaffected at 500 °C for pine (Fernandez et al. 2022). Aromatic condensation may increase stability while O-functionalisation may decrease it.

^j^Contradictory. Lab oxidative pyrolysis increases aromaticity (Park et al. 2019; Gao et al. 2024a, b, c), but wildfire charcoals show lower aromaticity (Santín et al. 2017), likely due to uncontrolled conditions.

^k^Simulated pyrolysis gas (20% CH_4_, 10% CO_2_, 70% N_2_) marginally increased fixed C and decreased H/C and O/C (Yan et al. 2020).

^l^CONTAMINANT RISK. Text discusses HCl, HF, hydrocarbon derivatives, and heavy metals in flue gases (Villeneuve et al. 2012).

## Environmental and economic considerations of pyrolysis atmospheres

The selection of pyrolysis atmosphere entails critical environmental and economic trade-offs that must be carefully evaluated alongside biochar property enhancements when evaluating its utilization for different applications.

The inert gases N_2_ and Ar are most commonly used in research studies, and N_2_ is also used in some industrial pyrolysis plants. However, the availability of compressed N_2_ in industrial applications is limited and would need to be acquired explicitly for this purpose, reducing the feasibility for large-scale operations. This is where alternative gases have advantages, as they can be available as part of the pyrolysis operation itself (flue-/pyrolysis gas) or as part of other industrial processes (CO_2_, steam, O_2_). This can bring significant economic, sustainability, and practical advantages utilizing existing resource streams.

Reactive gases, such as NH_3_ and O_2_, can substantially improve biochar functionality and hence use, for example, for sorption applications in water treatment, but also potentially for soil. However, they also introduce safety, handling, and potential emissions challenges. NH_3_, being toxic and corrosive, requires stringent containment and gas management protocols, increasing operational complexity and costs. Similarly, introducing O_2_ enhances exothermic oxidation reactions, potentially reducing biochar yield and increasing fire risk, which in turn affects process stability and the carbon sequestration potential.

Life cycle assessments (LCA) indicate that biochar systems' net greenhouse gas mitigation potential depends not only on biochar yield and stability but also on potential replacement of fossil fuel emissions and associated energy mix in the power grid (Lehmann et al. 2021). CO_2_-assisted pyrolysis integrated with industrial CO_2_ streams can improve process efficiency and reduce overall carbon footprint. However, elevated temperatures may lead to lower biochar yields due to enhanced gasification, making such temperatures less suitable for carbon sequestration (carbon dioxide removal) purposes. Steam atmospheres can increase liquid fuel yields but reduce char production, influencing the balance between energy recovery and carbon storage. Therefore, the use of a steam atmosphere must be carefully balanced, depending on whether the priority is energy efficiency or carbon sequestration.

From an economic perspective, the feasibility of alternative atmospheres depends on gas availability, cost, and infrastructure requirements. Integrating pyrolysis with existing industrial processes to utilize waste heat and flue gases offers promising pathways to reduce operational costs and improve sustainability. However, the increased capital and operational expenditures associated with handling reactive gases and ensuring safety must be offset by the higher value of engineered biochar tailored for specific high-value applications. Comprehensive techno-economic analyses and environmental impact assessments are essential to guide the selection and scale-up of pyrolysis atmospheres in commercial biochar production.

## Conclusions

Alongside peak temperature and feedstock composition, the pyrolysis atmosphere is one of the key parameters determining the yield and properties of biomass pyrolysis co-products. This review has demonstrated that the choice of pyrolysis atmosphere significantly influences the efficiency of the process and the characteristics of the resulting biochar, gases, and liquids. Specifically, non-inert atmospheres such as CO_2_, steam, and NH_3_ have shown promise in enhancing biochar functionality and reducing tar formation, offering a one-step process for producing engineered biochar.

Several critical research gaps remain that warrant further investigation. First, systematic experimental studies are needed to elucidate the mechanisms underlying the observed effects of different pyrolysis atmospheres. Second, comparative studies employing various reactor designs and operational parameters are required to optimize biochar production tailored to specific applications. Beyond these, exploring hybrid or sequential atmosphere strategies, where biomass undergoes staged pyrolysis with different gases introduced at defined phases, represents a promising avenue to optimize biochar yield and functionality synergistically.

The adoption of advanced reactor technologies, including microwave-assisted, plasma, and catalytic pyrolysis, offers further opportunities to harness novel atmosphere-process combinations, potentially improving energy efficiency and product characteristics. Importantly, most current knowledge derives from laboratory-scale studies; therefore, field-scale validation is urgently needed to assess the stability, performance, and environmental impacts of engineered biochar under real-world conditions.

Further research should also focus on integrating pyrolysis processes with industrial operations, leveraging waste gases and heat to improve overall energy efficiency and reduce carbon footprints. Standardization of experimental protocols and reporting, including detailed characterization of gas atmospheres, reactor configurations, and process parameters, is essential to improve reproducibility and comparability across studies. Integrating CO_2_ pyrolysis with industrial processes that produce CO_2_ and heat could enhance process efficiency and contribute to decarbonization efforts.

This review contributes new perspectives by highlighting the underexplored potential of alternative pyrolysis atmospheres and providing a comprehensive comparison of their impacts. By addressing the identified knowledge gaps and pursuing the suggested research directions, future studies can advance our understanding and application of biochar production, leading to more efficient, sustainable, and application-specific biomass conversion technologies.

## Supplementary Information

Additional file1 (DOCX 16 KB)

## Data Availability

The datasets used in this study are available from the corresponding author upon reasonable request.
