# Supplementary material for: Biochar production under different atmospheres: an overview
Source: Biochar. 2026 Jul 29;8(1):129. doi: 10.1007/s42773-026-00626-8 (PMC13421295; doi:10.1007/s42773-026-00626-8)
Supplement: Supplementary file 1 — Additional file1 (DOCX 16 KB) [file 42773_2026_626_MOESM1_ESM.docx]

Table S1. Mechanistic influences of process gases during biomass pyrolysis—dominant gas–solid interactions, indicative temperature windows, and first-order trends in product yields and char properties (reported relative to an N₂ baseline, holding other parameters constant).

| **Process gas** | **Dominant interactions (illustrative)** | **Typical temperature window (indicative)** | **Expected first-order trends (holding other parameters constant)** |
| --- | --- | --- | --- |
| N₂ (inert carrier) | Heat transfer; residence-time control | Any (process-dependent) | Baseline for comparison; secondary reactions driven by T and vapour residence time |
| O₂ / air (low % O₂) | Radical abstraction; partial oxidation of volatiles/char; exothermic heat release | >350–500 °C (strongly T-dependent) | ↓ Char yield; ↑ CO/CO₂; less heavy tars; can accelerate devolatilisation; possible increase in SSA via burn-off |
| CO₂ | Boudouard C + CO₂ ⇌ 2CO (etching); tar reforming | >700–800 °C (faster at higher T) | ↓ Char yield; ↑ SSA/micro-mesoporosity; ↑ CO; oils less oxygenated due to reforming |
| H₂O (steam) | C + H₂O ⇌ CO + H₂; tar steam-reforming; WGS | >700–800 °C (catalysis can lower) | ↓ Char yield; ↑ SSA; ↑ H₂/CO; reduction in heavy oxygenates in oils |
| H₂ | Radical capping; hydrogenation (limited without catalysts) | >500–700 °C (effects increase with T) | Slight ↔/↓ cross-linking; potential ↑ light hydrocarbons; modest changes to char unless catalytic surfaces present |
| CH₄ | Thermal cracking at very high T; potential carbon deposition | >900–1000 °C (non-catalytic) | Usually minor effects under typical biochar T; at high T: ↑ H₂, possible carbon deposition; limited direct etching |
| NH₃ | Surface nitridation; incorporation of N functionalities | >700–900 °C | N-doped char (pyridinic/pyrrolic/quaternary-N); altered basicity and adsorption; yield effect depends on severity. |

Notes:

a. Temperature windows are indicative. Onset and rates depend on reactor configuration (fixed/fluidised/auger), gas partial pressures and flows, heating rate, particle size, vapour residence time, and feedstock ash/metal catalysis.

b. Trends are first-order. Arrows (↑/↓/↔) denote increase/decrease/no consistent change across the literature relative to N₂ under matched feedstock and thermal history; exceptions occur where conditions differ.

c. Oxidative conditions refer to low-O₂ “oxidative pyrolysis” (typically ≤5–10 vol% O₂) rather than combustion; effects intensify with O₂ fraction and temperature.

d. Gas–solid reactions referenced include Boudouard (C + CO₂ ⇌ 2CO), steam gasification (C + H₂O ⇌ CO + H₂), water–gas shift (CO + H₂O ⇌ CO₂ + H₂), low-level oxidation (C + ½O₂ → CO/CO₂), and hydrogenation/capping of surface radicals. Naturally occurring or ash-derived metals (e.g., K, Ca, Fe, Ni) can lower apparent temperatures and alter selectivity.

e. Porosity terminology. “SSA” denotes BET specific surface area; micro/mesoporosity follow IUPAC definitions (micro <2 nm; meso 2–50 nm). Porosity changes arise from burn-off/etching, pore widening, and, at high severity, collapse/reordering.

f. Nitrogen functionalities. For NH₃ atmospheres, “N-doped char” indicates incorporation of pyridinic-, pyrrolic-, and graphitic/quaternary-N; distributions depend on temperature and residence time.

g. Methane caveat. CH₄ effects are generally minor under typical biochar conditions (≤800 °C) unless very high temperatures (≥900–1000 °C) or catalytic surfaces are present.

h. Scope. Table S1 summarises mechanistic expectations to orient readers; individual studies may deviate due to feedstock composition (cellulose/hemicellulose/lignin balance), mineralogy, and hydrodynamics.
